# Epigenetic Modifiers: Anti-Neoplastic Drugs With Immunomodulating Potential

**DOI:** 10.3389/fimmu.2021.652160

**Published:** 2021-03-30

**Authors:** Ken Maes, Anna Mondino, Juan José Lasarte, Xabier Agirre, Karin Vanderkerken, Felipe Prosper, Karine Breckpot

**Affiliations:** ^1^ Laboratory for Hematology and Immunology, Department of Biomedical Sciences, Vrije Universiteit Brussel, Brussels, Belgium; ^2^ Center for Medical Genetics, Vrije Universiteit Brussel (VUB), Universiteit Ziekenhuis Brussel (UZ Brussel), Brussels, Belgium; ^3^ Lymphocyte Activation Unit, Division of Immunology, Transplantation and Infectious Diseases, IRCCS San Raffaele Scientific Institute, Milano, Italy; ^4^ Immunology and Immunotherapy Program, Centro de Investigación Médica Aplicada, IDISNA, Universidad de Navarra, Pamplona, Spain; ^5^ Laboratory of Cancer Epigenetics, Centro de Investigación Biomédica en Red Cáncer (CIBERONC), Pamplona, Spain; ^6^ Hemato-oncology Program, Centro de Investigación Médica Aplicada, IDISNA, Universidad de Navarra, Pamplona, Spain; ^7^ Hematology and Cell Therapy Department, Clínica Universidad de Navarra, Universidad de Navarra, Pamplona, Spain; ^8^ Laboratory for Molecular and Cellular Therapy, Department of Biomedical Sciences, Vrije Universiteit Brussel, Brussels, Belgium

**Keywords:** epigenetics, cancer, immune evasion, tumor microenvironment, immunotherapy

## Abstract

Cancer cells are under the surveillance of the host immune system. Nevertheless, a number of immunosuppressive mechanisms allow tumors to escape protective responses and impose immune tolerance. Epigenetic alterations are central to cancer cell biology and cancer immune evasion. Accordingly, epigenetic modulating agents (EMAs) are being exploited as anti-neoplastic and immunomodulatory agents to restore immunological fitness. By simultaneously acting on cancer cells, e.g. by changing expression of tumor antigens, immune checkpoints, chemokines or innate defense pathways, and on immune cells, e.g. by remodeling the tumor stroma or enhancing effector cell functionality, EMAs can indeed overcome peripheral tolerance to transformed cells. Therefore, combinations of EMAs with chemo- or immunotherapy have become interesting strategies to fight cancer. Here we review several examples of epigenetic changes critical for immune cell functions and tumor-immune evasion and of the use of EMAs in promoting anti-tumor immunity. Finally, we provide our perspective on how EMAs could represent a game changer for combinatorial therapies and the clinical management of cancer.

## Highlights

1. Epigenetic mechanisms control the differentiation, function and memory of innate and adaptive immune cells.
2. Alterations in epigenetic mechanisms play a key role in tumor immune escape.
3. Epigenetic-targeted therapy increases tumor immunogenicity and triggers anti-tumor immunity.
4. Epigenetic-targeted therapy adds significant value to existing cancer immunotherapies, including vaccination, adoptive T cell therapy and immune checkpoint inhibition.

## Introduction

Studies on the epigenome and chromatin states of cancer cells showed several vulnerabilities that can be exploited for therapy. Initial studies however, mostly limited their analysis to cancer cells, ignoring the tumor microenvironment (TME) in which cancer cells are embedded. The TME comprises the tumor stroma, blood and lymphatic vessels, infiltrating inflammatory cells and a variety of associated tissue cells. The continuous cross talk of the TME with proliferating tumor cells creates a unique and heterogeneous environment critical for the growth of the tumor and response to therapy. It is increasingly appreciated that epigenetic alterations, which occur both in tumor cells and in immune cells within the TME (such as CD11b^+^ myeloid cells, CD4^+^ and CD8^+^ lymphoid cells), further increase the complexity within tumor tissue and represent major determinants of cancer cell growth, immune evasion and drug resistance (1, 2). This knowledge has instigated research into the use of epigenetic modulating agents (EMAs) to manipulate both the tumor and the TME, and as such induce tumor regression.

Epigenetics is an umbrella term given to all the processes that mediate changes in gene expression without altering the DNA code [reviewed in (3)]. The most studied epigenetic mechanisms include post-translational histone modifications and DNA methylation.

Post-translational histone modifications occur in the N-terminal regions of histone tails and include methylation, acetylation, phosphorylation, sumoylation and ubiquitination of lysine, arginine, serine, threonine and tyrosine residues (4). Of these, acetylation and methylation of distinct lysine residues of histone tails have been abundantly studied. Histone acetylation and de-acetylation in lysine residues are mediated by histone acetyltransferases (HAT) and histone deacetylases (HDAC), respectively. Histone acetylation in lysine residues is a marker associated with active gene transcription, as acetylated histone tails open up chromatin resulting in recruitment of the transcriptional machinery (4). Histone methylation or demethylation of lysine and arginine residues is catalyzed by histone methyltransferases (HMT) or histone demethylases (HDM) (4). The outcome of histone methylation on transcription is dependent on the level of methylation, the modified amino acid and its position. For example, histone 3 lysine 9 or lysine 27 trimethylation (H3K9me3, H3K27me3, respectively) are modifications associated with transcriptional repression while histone 3 lysine 4 trimethylation (H3K4me3) and histone 3 lysine 36 trimethylation (H3K36me3) are markers associated with active euchromatin and transcriptional elongation, respectively. Moreover, histone modifications can be specifically found in certain genomic regions. For instance, H3K4me3 is most commonly associated with promoter regions, while H3K4me1 is a marker for enhancers. H3K27ac serves as an activation marker of both promoters and enhancers (5). Equally important are enzymes that recognize or read these histone modifications. Proteins that contain bromodomains or chromodomains recognize these methylated or acetylated residues, respectively. These proteins are recruited to histones and facilitate the formation of protein complexes involved in DNA replication and repair, gene expression and genome integrity (4). Of note, many of the enzymes responsible for histone post-translational modifications also modify non-histone proteins, thereby influencing their activation, protein-binding properties, degradation and stability (6).

DNA methylation entails the addition of a methyl group (CH_3_) on cytosine (5mC) in CpG dinucleotides. DNA methyltransferases (DNMT) 3A and 3B mediate *de novo* DNA methylation, while DNMT1 maintains existing DNA methylation patterns. Passive DNA demethylation occurs when DNMT1 does not methylate cytosine residues during replication. Active demethylation of DNA is catalyzed by Ten-Eleven-Translocation enzymes TET1, TET2 and TET3, which convert DNA methylated cytosine into hydroxymethylcytosine, formylcytosine and carboxycytosine after which the modified cytosine is removed through base-excision repair and changed into non-methylated cytosine. CpG methylation is found in genomic repetitive elements that contribute to genome stability. Moreover, DNA methylation induces gene silencing of neighboring genes when densely clustered CpGs or “CpG islands” located in promoter or enhancer regions are hypermethylated. DNA methylation of gene bodies and transposable element also represent a level of regulation of gene expression and splicing, although additional studies are currently needed to better elucidate their functional contribution (7).

Specific epigenetic modulating agents (EMA) have been identified and tested for their anti-tumor effect, or their ability to improve sensitivity of tumor cells to radio-, chemo- and even immunotherapy. The first generation of EMAs mainly targeted one specific class of epigenetic enzymes, such as DNMT inhibitors (e.g. Azacytidine and Decitabine) and HDAC inhibitors (e.g. Vorinostat and Panobinostat). However, epigenetic processes and their effect on gene regulation are the result of a coordinated interaction between different epigenetic alterations. For this reason, combined inhibition of, e.g., DNMTs and HDACs has been studied in several different cancer models with profound immune-related effects (8–11). As a more innovative approach, a novel class of compounds with dual inhibitory activity is gaining considerable attention. For example, the HMT/DNMT1 dual inhibitor CM-272 targets the HMT G9a and DNMT1, and has proven concomitant anti-neoplastic effects and immunomodulation (12, 13). In the first chapter, we review some of the epigenetic events critical to the behavior of several immune cell types in the context of cancer (**Supplementary Table S1**). In the last two chapters, we review recent findings in the exploitation of EMAs (i) to boost tumor immunogenicity and (ii) in immunotherapeutic strategies.

## The Immune Cell Epigenome in the Cancer Microenvironment

In cancer, the equilibrium between lymphoid and myeloid cell responses is often perturbed. The increase in immature or dysfunctional myeloid cells is accompanied by a reciprocal decline in the quantity and/or quality of the lymphoid response. Characteristic myeloid cell populations are tumor associated macrophages (TAM), tumor associated dendritic cells (TADC) and immature myeloid derived suppressor cells (MDSC). These govern the efficacy of CD8^+^ cytotoxic T lymphocytes (CTL), mostly hindering their tumor cell killing activity. Given their importance in the TME, we have reviewed their epigenetic regulation and discussed the implications thereof in the context of cancer with the aim of understanding which epigenetic targets in immune cells could represent suitable targets to promote anti-tumor immunity.

### The Epigenome of TAM

TAM are abundantly present in many cancer types, representing a diverse population of mixed ontogeny, derived from monocytes or embryonic precursors, with opposed polarization states (14). Classically activated and alternatively activated TAM, also referred to as M1 and M2 TAM respectively, represent two extremes of a dynamic changing state of macrophage polarization (15). While M1 TAM promote a pro-inflammatory environment and protective T helper (T_H_) 1 and CTL responses, M2 TAM favor T_H_2 polarization, tumor progression and dissemination. M2 TAM can suppress anti-tumor immune responses, promote tumor angiogenesis and enable cancer cells to disseminate at distant sites where they can support cancer cell survival and growth into metastatic lesions (16). In addition, M2 TAM have been shown to counteract the anti-tumor effects of chemotherapy, radiation therapy, targeted therapy, and immunotherapy, as extensively reviewed elsewhere (17).

**Figure 1:**
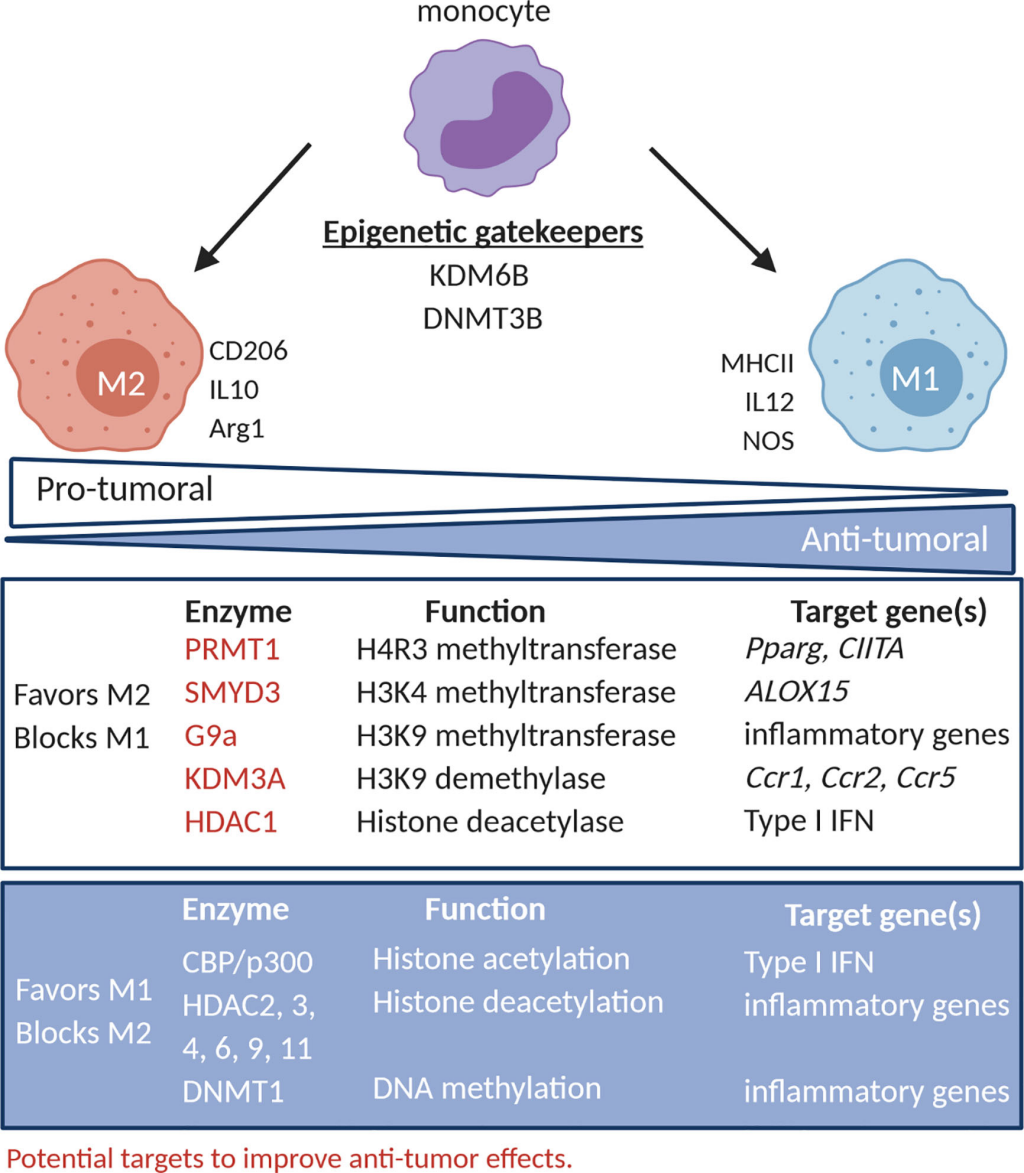
TAM polarization and its epigenetic regulatory networks. The activity of epigenetic regulators has been correlated to the phenotype and function of TAM. PRMT1, SMYD3, G9a (HMT) KDM3A (HDM) and HDAC1 promote TAM with an M2-like phenotype (CD206) and function (IL10 and arg-1), while CBP/p300 (HAT), HDAC2, 3, 4, 6, 9, 11 and DNMT1 promote TAM with an M1-like phenotype (MHC-II) and function (IL12, NOS). DNMT3B and KDM6B (HDM) seem to act as gatekeepers and can in certain conditions promote M1- or M2-like behavior. PRMT1, SMYD3, G9a, KDM3A and HDAC1 represent potential targets to improve increase the M1/M2 balance. HMT, histone methyltransferase; HDM, histone demethylases; HAT, histone deacetylase; HDAC, histone deacetylases.

Understanding the epigenetic modifications responsible for M1 versus M2 polarization in the TME is critical to instruct the use of EMAs to offset the tumor promoting effects of M2 TAM. With this aim in mind, in the following paragraph, we have discussed epigenetic modifications linked to TAM polarization (**Figure 1**).

#### Histone (de)methylation

HMT, such as protein arginine N-methyltransferase 1 (PRMT1) and MYND domain containing 3 (SMYD3), have been described to favor M2 polarization and, as such, represent targets to inhibit accumulation of tumor-promoting TAM (18–24). PRMT1 was shown to be responsible for histone 4 arginine 3 methylation (H4R3me) in the promoter of peroxisome proliferator activated receptor-γ (*Pparg*), reducing *Pparg* expression in interleukin 4 (IL4) stimulated mouse macrophages, thereby promoting M2 polarization (18). Accordingly, the PRMT1 inhibitor, AMI-1, reduced IL4-induced *Pparg* expression in mouse macrophages (18), abrogated the ability of THP1 macrophages to ingest apoptotic bodies, and reduced M2 polarization in alcohol-induced hepatocellular carcinoma (20). Moreover, PRMT1 was shown to negatively regulate M1 polarization in interferon γ (IFNγ) stimulated RAW264.7 cells by repressing class II major histocompatibility complex transactivator (CIITA) (19), further suggesting that PRMT1 inhibition favors an anti-tumoral M1/M2 ratio. The expression of the H3K4 methyltransferase SMYD3 was induced in human macrophages exposed to IL4, resulting in transcriptional activation of *ALOX15*, a lipoxygenase M2 marker (21). Also, the H3K9me2 HMT G9a (or EHMT2), has been implicated in macrophages tolerization, leading to unresponsiveness to M1 polarizing stimuli like lipopolysaccharide (LPS) (25). Mechanistically, G9a interacts with several transcription factors, among which ATF7 and NF-κB family members, resulting in G9a recruitment to specific loci to deposit H3K9me2, leading to repression of inflammatory gene expression (25–27).

Concerning HDM, evidence is in place that KDM6B (jumonji D3 [JMJD3]) is a gate keeper of macrophage polarization. KDM6B was shown to be responsible for expression of typical M2 markers, like interferon regulatory factor 4 (IRF4), arginase-1 (Arg-1), and CD206, in mouse macrophages stimulated with IL4 and/or IL13 (22, 24). Also, KDM6B was shown to positively regulate pro-inflammatory genes in LPS-stimulated mouse macrophages independent of its demethylation activity (23). Accordingly, inhibition of KDM6B by GSK-J4 molecule, reduced both CD206 expression in IL4-stimulated human macrophages and repressed M1 inflammatory cytokines (e.g. tumor-necrosis factor alpha [TNFα]) in LPS- or IFNγ-stimulated human macrophages (28, 29). To date, it remains to be determined whether targeting KDM6B or H3K27 methylation level represent a valuable therapeutic opportunity. Likewise, the H3K9 HDM KDM3A, has also been shown to impact on the epigenetic status of macrophages (30). Hypoxia-dependent inhibition of KDM3A and the resulted increase the level of the H3K9me2/3 repressive histone mark in the promoter regions of C-C motif chemokine ligand 2 (*Ccl2*), C-C motif chemokine receptor 1 *(Ccr1*), and *Ccr5* hindered their expression in mouse macrophages and RAW264.7 cells (30). Likewise, in HeLa and A673 xenografts, KDM3A expression is induced by hypoxia and nutrient starvation. Hence, siRNA mediated KDM3A inhibition resulting in anti-tumor activity, best explained by reduced infiltration of CD11b^+^ macrophages and angiogenesis (31). As TAM in hypoxic regions mainly exhibit an M2 polarization state (32), it is tempting to speculate that hypoxia and KDM3A control macrophage function at various levels.

#### Histone (de)acetylation

Histone lysine (de)acetylation also regulates macrophage polarization, albeit with somewhat contradictory evidence. Virus-induced type I IFN gene induction in macrophages requires a transition from a HDAC containing repressor complex to a HAT (CBP/p300) containing activation complex (33). However, inhibiting HAT with Anacardic Acid induced phagocytosis, migration and secretion of nitric oxide, IL6 and TNFα in primary peritoneal macrophages (34). Thus, further studies are needed to elucidate the role of HAT in controlling macrophage responses. Moreover, histone deacetylation or augmented expression of class I, II and IV HDAC may be associated with a favorable balance of M1 over M2 macrophages in the TME as several studies showed that HDAC2, 3, 4, 9 and 11 are needed for inflammatory gene expression and prevention of M2 polarization (35–38). However, the pan-HDAC inhibitor Vorinostat inhibited TAM infiltration in estrogen receptor negative PyMT mammary tumors, thereby delaying tumor growth (39, 40), and inhibited tobacco smoke-related increase of F4/80^+^ Arg-1^+^ M2-like macrophages in a murine KRAS-driven pancreatic cancer model (39, 40). Thus histone modifying enzymes play a complex role in macrophage biology and suggest that rather than pan-HDAC inhibitors, more specific targeting prove more effective. As an example, specific targeting of HDAC1 instructed a pro-inflammatory macrophage phenotype, while inhibition of HDAC6 inhibited pro-inflammatory signaling and promoted an anti-inflammatory phenotype (41).

#### DNA (de)methylation

With respect to DNA methylation, experiments using RAW264.7 and mouse macrophages suggested a role for DNMT3B as a gatekeeper of macrophage differentiation (42). Indeed, knock-down of DNMT3B resulted in M2 polarization and M2 markers induction independently of IL4, while repressing LPS-induced TNFα secretion and CCL2-dependent migration (42). Cheng et al. instead identified a role for DNMT1 in macrophage polarization. These authors showed that DNMT1 stimulates release of pro-inflammatory cytokines through DNA hypermethylation and reduction of expression of SOCS1, a negative regulation of the JAK2/STAT3 pathway (43). However, the combination of the DNMT1 inhibitor, Azacytidine, with an irreversible inhibitor of polyamine biosynthesis, α-di-fluoromethylornithine, increased M1 macrophages in the TME of an ovarian cancer model (44). Thus, to date also DNMT appear to play pleiotropic functions in TAM biology.

Overall, available evidence indicates that EMA would help skew the M1/M2 ratio in the TME. Pan-HDAC inhibitors have promising effects, yet testing specific inhibitors, such as HDAC1 inhibitors, might be more interesting. Likewise, PRMT1 and KMD3A targeting might shift the balance of macrophage polarization towards M1. Thus, further investigation is needed to better define the impact of EMA on TAM and their impact on anti-tumor immunity.

### The Epigenome of TADC

Different subsets of dendritic cells (DC) have been described in mouse and human, and differ in their ontogeny, phenotype and function as reviewed elsewhere (45, 46). Known subsets include plasmacytoid DC (pDC), conventional DC (cDC), subdivided in type 1 (cDC1) and type 2 (cDC2), and monocyte derived DC (moDC). DC can induce adaptive immune responses to foreign antigens, including tumor antigens (47). However, cues in the TME can abrogate functional differentiation and activation of TADC and as such undermine stimulation of anti-tumor T cell immune responses (48). cDC1 and cDC2 develop from committed bone marrow progenitor cells as two functionally distinct subsets; XCR1^+^ IRF8^+^ cDC1 and CD11b^+^ IRF4^+^ cDC2 (49). In both mice and humans, cDC1 express high levels of the chemokine receptor XCR1 and the C-type lectin endocytic receptor CLEC9A (50), and are reported to stimulate CTL, natural killer (NK) cells and NKT cells, therefore have an anti-tumor role (51). This is corroborated by the observation that enrichment of cDC1 in human tumors is associated with a good prognosis in several cancer types (51–55). Identifying cDC2 is more challenging as canonical markers are not yet defined. Therefore, cDC2 are identified, based on the expression of CD11b, CD11c, MHC-II, CD172a, and CD47 (SIRPα) (mice) or CD1c (BDCA-1) (humans) (56). This DC-subset is implicated in activation of T_H_17 responses, a T_H_ subset that has been correlated with good and bad prognoses in cancer (57). Moreover, similar to moDC, cDC2 exert CTL-suppressive activities (e.g. through L-arginine metabolism) (58), further implicating cDC2 in tumor promotion. While pDC are less abundant in tumors and mainly considered to act as producers of type I IFN upon viral infection, they should not be disregarded. In mice, pDC are characterized as CD11c^low^ MHC-II^+^ B220^+^ Siglec-H^+^ cells, while their human counterparts are characterized as CD123^+^ CD33^-^ (46, 59). In contrast to cDC1, however, similar to cDC2, the presence of pDC is not a positive prognostic factor in several human cancer types (60–62). Nonetheless, in mouse models of breast cancer, activated pDC can directly kill tumor cells through TNF-related apoptosis inducing ligand (TRAIL) and granzyme B (GzmB) as well as they jumpstart activation of CTL and NK cells (63). Lastly, tumor-associated moDC are derived from Ly6C^+^ (mice) or CD14^+^ (humans) monocytes and are characterized as CD11c^+^ MHC-II^+^ F4/80^−^ CD64^+^ in mice and CD14^+^, sometimes CD16^+^ in humans (64, 65). Ex-vivo derived MoDC have been shown to serve as potent anti-tumor vaccines but have also been described as suppressors of anti-tumor immunity (45, 66).

**Figure 2:**
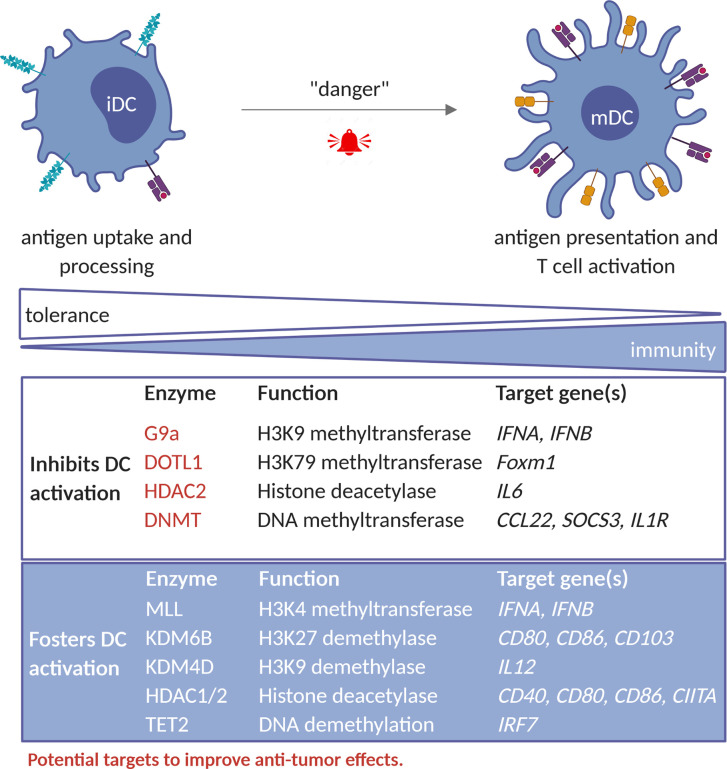
Epigenetic factors that regulate TADC activity. Epigenetic enzymes that foster DC-activation include MLL complex (HMT); KDM6B and KDM4D (HDM); HDAC1 and 2 as well as TET2. Collectively these enzymes regulate the expression of genes involved in antigen presentation and stimulation of CTL-responses, such as *CIITA, CD40, CD80, CD86, IL12 and IFNA/B*. In contrast, enzymes such as the G9a (HMT), HDAC2, and the DNMT1, DNMT3A and DNMT3B rather inhibit DC-activity by acting on genes such as *IL6, IFNA/B, CCL2, SOCS3 and IL1R*. G9a, DOTL1, HDAC2 and DNMT represent potential targets to improve DC mediated anti-tumor immunity. iDC, immature dendritic cell; mDC, mature dendritic cell; HMT, histone methyltransferase; HDM, histone demethylases; HDAC, histone deacetylases.

As in the case of TAM, understanding the epigenetic regulation of DCs in the context of cancer might instruct the use of EMAs to rewire the TADC’s anti-tumor properties (**Figure 2**).

#### Histone (de)methylation

Several studies underlined the role of epigenetic events in controlling DC development and function (67, 68). For instance, bacterial infection was causally linked to an increase in H3K4me1 and H3K27ac and DNA hypomethylation at enhancer regions of inflammation-related genes (69). Also the induction of a type I IFN response upon viral infection is regulated by HMTs, in particular the MLL complex and G9a, although with opposite effects. While MLL induced H3K4me3 levels favoring type I IFN gene expression, G9a induced H3K9me2 deposition and repressed *IFNA* and *IFNB* expression (70, 71). Of interest, G9a inhibition restored expression of *Ifna*, *Ifnb* and IFN-stimulated genes (ISGs) in murine bone marrow-derived DC (70), supporting the possibility that inhibition of G9a could be attempted to promote TADC anti-tumoral activity. During LPS activation of DCs, the histone demethylases KDM6B (JMJD3) and KDM4D (JMJD2D) have been shown to erase the repressive marks H3K27me3 and H3K9me3, thereby upregulating co-stimulatory and pro-inflammatory genes and stimulating inflammation. Moreover, inhibition of these enzymes relieved autoimmune reactions in mice (72, 73). Therefore, intratumoral activation of KDM6B or KDM4D would be interesting to promote local DC activity.

Upon TGFβ-mediated moDC differentiation, dynamic changes in active H3K4me3 and repressive H3K27me3 marks, catalyzed yet to be defined enzymes, take place. This led to transcriptional upregulation of co‐stimulatory molecules and cytokines/chemokines, and downregulation of differentiation markers, respectively (74). In case of pancreatic and colon cancer, FOXM1 has an immunosuppressive role through impairment of DC maturation and T cell responses. These immune suppressive effects are partially restored by targeting DOTL1 using EPZ004777 molecule, as this results in decreased histone 3 lysine 79 dimethylation (H3K79me2) in the *Foxm1* locus and decreased *Foxm1* expression (75), suggesting that DOTL1 may be an interesting target to improve DC function.

#### Histone (de)acetylation

Only a few studies have investigated the role of histone lysine (de)acetylation in DC. For instance, histone lysine acetylation was reported to regulate the expression of MHC-II and *Arg1* during DC differentiation from bone marrow cells (76, 77). Choi et al. found that GM-CSF increased H3 and H4 lysine acetylation in the CIITA promoter and increased STAT5 binding during DC differentiation (76). In the case of pDC, inhibition of HDAC decreased PU.1 expression and suppressed the recruitment of PU.1 to *FLT3* and *IRF8*, critical for pDC differentiation (78).

Together, available literature suggest that HDAC have context dependent roles during DC differentiation and in general promote DC function (79). In agreement, Panobinostat, a pan-HDAC inhibitor (HDACi), hindered antigen uptake and presentation, expression of co-stimulatory proteins and pro-inflammatory cytokine as well as T cell stimulatory capacity of DC (80). Thus, HDAC inhibitors might not be suited to improve DC functions at tumor sites.

#### DNA (de)methylation

DNA methylation also plays a role in DC function. DNA demethylation can occur in response to IL4, which is needed for DC differentiation from monocytes (81). Loss of DNA methylation is observed in upon maturation of DC followed by *de novo* methylation and is attributed to modulation of expression of DNMT1, 3A and 3B (82). Likewise, induction of type I IFN after viral infection required TET2-dependent DNA demethylation of the *IRF7* gene in pDC (83). Therefore, DNA hypomethylating agents are of interest for stimulation of pro-inflammatory DC functions. Accordingly, Azacytidine was reported to upregulate the expression of CD40 and CD86 and to hinder expression of anti-inflammatory IL10 and IL27 in monocytes-derived human DC (84). These events have been linked to increased IL17 and reduced IL4 expression in CD4^+^ T cells in Azacytidine-treated acute myeloid leukemia (AML) and myeloid dysplastic syndrome (MDS) patients (84). In line with this notion, a combination of Azacytidine, IFNγ and the HDAC1 and HDAC2 inhibitor, romidepsin, induced IFN signaling in IL4-induced human DC and increased their migratory capacity (85).

To summarize, available evidence suggests that G9a, DOTL1 and DNMT might be promising targets to improve DC function in the TME, while the KMD6B, KMD4B and HDAC would not. Future studies would be needed to corroborate existing evidence and define most suitable combinations.

### The Epigenome of MDSC

**Figure 3:**
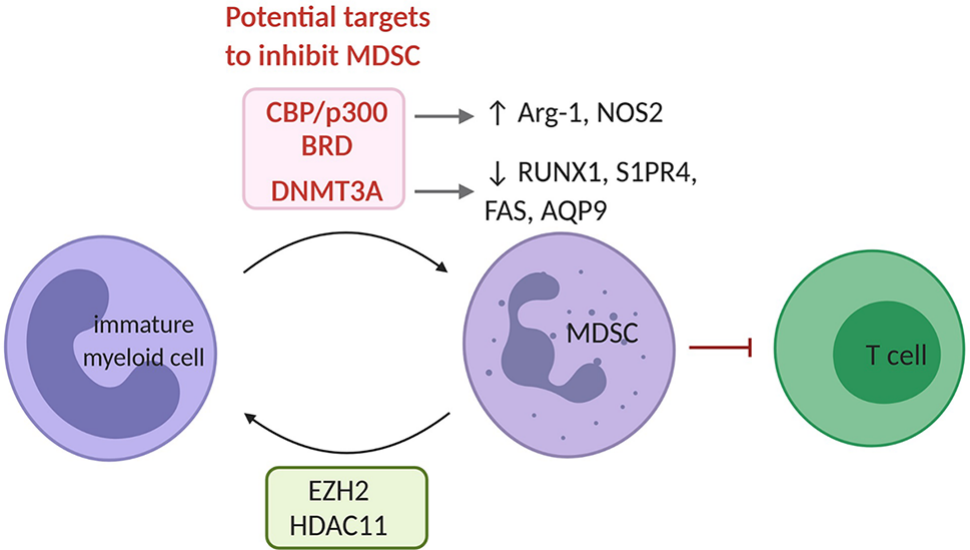
The epigenetic landscape of MDSC. EZH2 and HDAC11 serve as negative regulators of MDSC differentiation from immature myeloid. In MDSC, the bromodomain (BRD) of CBP/p300 acts as a critical regulator of H3K27Ac across promoters and enhancers of pro-tumorigenic target genes such as Arg-1 and NOS2. Also, increased DNMT3A levels have been shown in MDSC and have been linked to repression of immunity-related genes such *RUNX1, S1PR4, FAS* and *AQP9*. CBP/p300 and its bromodomain or DNMT3A represent potential targets to inhibit MDSC. MDSC, myeloid derived suppressor cell; HDAC, histone deacetylase.

MDSC are a collection of diverse myeloid cells of granulocytic (G) or monocytic (M) origin that share the functional trait of suppressing immune effector cells (17, 86). The generation and egress of MDSC from bone marrow to blood and their subsequent infiltration into the tumor is promoted by pro-inflammatory mediators, growth factors and chemotactic factors that are produced by cancer cells. Overall, MDSC are characterized by expression of various enzymes that endow them with the ability to suppress anti-tumor T cell responses. These enzymes include Arg-1, indoleamine 2,3 deoxygenase (IDO), and inducible nitric oxide synthase. Moreover, MDSC assist in recruiting other immunosuppressive cells, such as TAM, TADC and regulatory T cells (Treg), thereby enforcing the immunosuppressive TME (87, 88). The epigenetic regulation of MDSC has been less studied, however, there are several studies showing that epigenetic mechanisms are involved in MDSC differentiation, function and survival (**Figure 3**).

#### Histone (de)methylation

The inhibition of the H3K27me3 HMT EZH2 using GSK126 increased the presence of MDSC within the TME of LLC (Lewis lung carcinoma) and MC38 (colon carcinoma) models. This resulted from a differentiation of MDSC from hematopoietic progenitor cells upon inhibition of EZH2. Co-treatment with agents that deplete MDSC such as gemcitabine and 5’-Fluoroacil, restored the anti-tumor effects of GSK126 (89). EZH2 thus functions as a negative regulator of MDSC development, therefore does not represent a suitable target to tackle MDSC. This is also supported by the reduction of inflammation in inflammatory bowel disease upon EZH2 inhibition (90).

#### Histone (de)acetylation

The contribution of histone lysine (de)acetylation to MDSC development has so far been poorly characterized. HDAC2 has been shown to skew myeloid differentiation to G-MDSC rather than DC or TAM in the EL4 (lymphoma) model (91). In contrast, HDAC11 was reported to negatively regulate MDSC development *in vivo* (92). The HAT CBP/p300 and its bromodomain, by controlling H3K27Ac in promoters and enhancers of pro-tumoral genes, was found to promote the MDSC’s suppressive function. Its inhibition hindered their suppressive activity in the CT26 (colon carcinoma) model (93). However, the use of inhibitors has been found to elicit controversial effects. Recently, the HDACi, Valproic Acid, was shown to reduce the MDSC immunosuppressive function both *in vitro* and *in vivo* (94). Moreover, the HDACi Vorinostat depleted MDSC in 4T1 mammary tumors (95). Likewise, the class I HDACi Entinostat, was reported to have anti-tumor effects by neutralizing MDSC through epigenetic reprogramming in mouse models of pancreatic, breast, lung and renal cell cancer (96, 97). On the contrary, HDACi Trichostatin-A favored GM-CSF-induced expansion of MDSC *in vitro* and *in vivo* (98). Thus, further studies will have to better define the function of specific HAT and HDAC in MDSC biology to define their targeting in therapeutic treatments.

#### DNA (de)methylation

The functions of MDSC are linked to specific alterations in DNA methylation patterns catalyzed by DNMT3A in response to tumor cell-related factors. DNMT3A downregulation erased the MDSC specific DNA methylation patters and blocked their immunosuppressive capacity (99). Likewise, the DNA demethylating agent Decitabine reduced the number of bone marrow-derived MDSC in the 5T33 and the MPC11 multiple myeloma (11, 100), and a murine leukemia model where it also promoted the efficacy of adoptive T cell transfer (101). Of interest, in renal (Renca), colon (CT26) and prostate (TRAMP-C2) carcinoma models, Decitabine promoted MDSC differentiation into CD11c, MHC-II and CD86 competent antigen presenting cells, capable of protecting naïve mice from tumor challenge (102). Thus, DNMTs appear suitable targets to overcome the immunosuppressive MDSC potential in the TME.

So far, DNA methylation appears to be the most promising epigenetic alteration to target MDSC in the TME. Whether additional EMA or even combinations of EMA targeting different enzymes would provide a further benefit remains to be tested.

### The Epigenome of T Cells

CD4^+^ and CD8^+^ αβT cells derive from thymic precursors. Upon activation and differentiation into effector and memory T cells subsets within secondary lymphoid organs, they jointly contribute to tumor immune surveillance. The interplay of signals generated by the TCR, co-stimulatory and cytokine receptors lead to the activation of a plethora of transcription factors and of epigenetic mechanism, which jointly instruct cell differentiation and cell fate (103).

**Figure 4:**
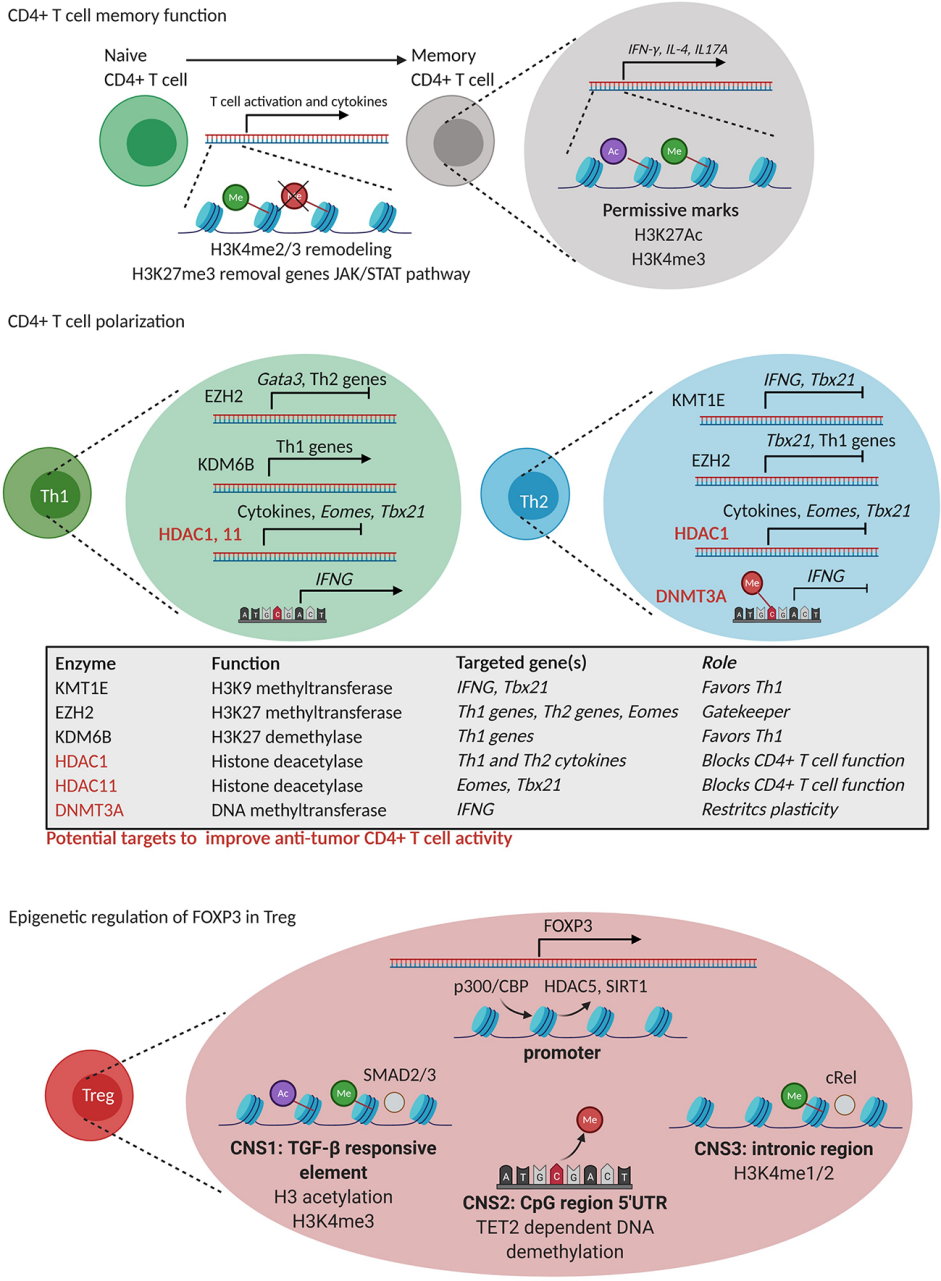
Epigenetic regulation of CD4^+^ T cell differentiation. **CD4^+^ T cell memory formation**: During memory formation, remodeling of H3K4 methylation (green) marks takes place together with the removal of repressive H3K27me3 (red) marks in genes of the JAK-STAT pathway. Moreover, permissive H3K4me3 (green) and H3K27Ac (purple) marks are present in genes related to CD4^+^ T cell function. **CD4^+^ T cell polarization**: T_H_1 polarization is controlled by EZH2 that silences T_H_2-related genes and *vice versa*. KDM6B favors T_H_1 polarization by positive regulation of T_H_1-related genes while KMT1E negatively regulates *IFNG* and *Tbx21* (T-bet) gene expression. HDAC1 and 11 are general inhibitors of CD4^+^ T cell function through silencing of cytokine production, *Eomes* and *Tbx21* (T-bet). **Epigenetic regulation of FOXP3 expression in Treg**: FOXP3 expression is regulated by epigenetic processes in Treg including (i) exchange of repressive HDAC5 and SIRT 1 for a permissive CPB/p300 (HAT) complex, (ii) permissive H3K4me3 (green) and H3 lysine acetylation (purple) in a TGFβ response element (CNS1), (iii) TET2 dependent DNA demethylation of a 5’UTR region (CNS2), and (iii) H3K4me1/2 poised state of CNS3 needed for *Foxp3* expression. HDAC1, 11 and DNMT3A represent potential targets to improve CD4^+^ T cell anti-tumor immunity.

CD4^+^ T cells can be divided in regulatory T cells (Treg) and T helper (T_H_) cells, among which T_H_1, T_H_2, T_H_17 and T_FH_ with various effects on tumor immunity. In particular, T_H_1 cells assist in protective antitumor immune responses as they support the differentiation of CD8^+^ T cells into CTL, able to recognize tumor antigen-derived peptides in the context of MHC-I and instruct tumor cell killing (104). In contrast to T_H_1 cells and CTL, T_H_2 and Treg represent CD4^+^ T cell subsets that facilitate tumor progression by exerting immunosuppressive activities within the TME. Histone modification and DNA methylation control both the process of memory establishment and of subsets differentiation of which we summarized several critical events in the following paragraphs (**Figure 4**).

#### Epigenetics in CD4^+^ T Cell Differentiation

##### Memory Function

The comparison of the epigenome of human naive and memory CD4^+^ T cells revealed that histone modifications, together with DNA methylation, play a complex interplay in memory CD4^+^ T cell formation, and supported a linear model of differentiation (**Figure 4**) (105). For instance, chromatin immunoprecipitation (ChIP)-sequencing analysis revealed dynamic changes in the activation marks H3K4me2 and H3K4me3 and in the expression of genes involved in naive and memory human CD4^+^ T cell activation and in cytokine gene expression (106). Likewise, early after activation of human naive and memory CD4^+^ T cells, H3K27me3 demethylation was observed in genes of the JAK-STAT pathway, such as *JAK2* and *IL12RB2* (107), by means of KMD6B in both naive and memory human CD4^+^ T cells. Moreover, the presence of permissive epigenetic marks such as H3K4me3 and H3K27Ac in promoter and enhancer regions of signature genes such as *IFNG, IL4* and *IL17A* has been shown to account for fast memory responses upon secondary antigen encounter by human memory CD4^+^ T cell function (108).

##### Polarization

Polarization of CD4^+^ T cells into different T_H_ subsets is mediated by joint interactions of DNA methylation and histone modifications that functionally instruct different enhancers and transcription factors to regulate expression of lineage specific cytokines and receptors (**Figure 4**) (109–111). As an example, Adoue et al. showed very recently that the HMT KMT1E favors T_H_2 polarization through deposition of the repressive mark H3K9me3 in cis-regulatory of T_H_1 specific genes such as *IFNG* and *Tbx21* (encoding T-bet) (112). In murine cells, also the HMT EZH2 controls the T_H_1 and T_H_2 differentiation by depositing the repressive mark H3K27me3 at the promoter of the T_H_1-promoring transcription factor *Tbx21* in T_H_2 cells, *and vice versa*, at the promoter of the T_H_2-promoting transcription factor *Gata3* in T_H_1 cells (113). Moreover, in the absence of polarizing signals, EZH2 was also reported to suppress *Eomes*, thereby repressing the spontaneous formation of IFNγ producing T_H_1 cells (113). Given the role of EZH2 in early CD4^+^ T cell differentiation, its targeting should be further investigated as a strategy to modulate CD4^+^ T cell responses during tumor immunity. The H3K27me3 HDM, KDM6B, has also been implicated in T_H_1 differentiation through the induction of T_H_1 related transcription factors and cytokines (114). Nevertheless, the loss of KDM6B resulted in T_H_2 and T_H_17 differentiation (114), indicating that the targeting of KDM6B might not improve anti-tumor CD4^+^ T cell responses.

In addition to histone methylation, histone (de)acetylation also regulates T_H_ cell functions. For instance, HDAC1 was shown to repress T_H_1 and T_H_2 effector functions by downregulating cytokine production (115). Similarly, HDAC11 was shown to repress *Eomes* and *Tbx21* expression hindering *Ifng* and *Il2* expression in T_H_1 cells (116). This would suggest that inhibition of HDAC1 and HDAC11 might be attempted to promote CD4^+^ T cell function at the tumor site (**Figure 4**).

DNA methylation has also been implicated in T_H_ cell differentiation. A rapamycin-sensitive signal downstream of the TCR and the CD28 costimulatory receptor was found to acutely control DNA methylation within proximal promoter regions of *Ifng*, *Il4* and *Foxp3*, hence controlling CD4^+^ T cell differentiation (117). In addition, DNMT3a and *de novo* DNA methylation were shown to restrict T helper plasticity by silencing regulatory regions of the *Ifng* gene (118). Accordingly, CpG residues within the *Ifng* promoter were found to become hypermethylated during the *in vitro* differentiation of mouse naıve T cells into T_H_2, but not T_H_1 cells (119). Thus, the role of DNMTs is very similar to the role of EZH2, as described above, suggesting that a deeper understanding of the role of *de novo* DNA methylation of CD4^+^ T cell function in tumors is warranted.

#### Treg Development and Epigenetics

Epigenetic changes in Treg merit attention, as Treg play an immunosuppressive role in the TME. Treg can be generated during thymic development at which time they are referred to as natural Treg (nTreg) or can be generated in the periphery when naïve CD4^+^ T cells are activated in the presence of TGFβ and/or IL10 at which time they are referred to as inducible Treg (iTreg). ChIP assays revealed an increase in H3K9/14Ac and H3K4me3 and a reduction of H3K27me3 in the promoter of the genes encoding the cell surface molecules *Il2ra* (CD25), *Ctla4* (CD152), *Nt5e* (CD73), *Icos* (CD278), and the transcription factor *Ikzf2* (Helios), all upregulated in Treg (120). Several studies have identified cell specific super-enhancers, accompanied by a strong activation linked histone modification, open chromatin states, strong binding of transcription factors, and increased DNA demethylation (121–123). Therefore, there is a potential therapeutic window for modulation of Treg activity in cancer through epigenetic therapy. Pharmacological inhibition of G9a methyltransferase activity in conventional T cells promotes T_H_17 and Treg differentiation, suggesting that G9a-dependent H3K9me2, a repressive histone modification during T cell differentiation, is a homeostatic epigenetic checkpoint that regulates T_H_17 and Treg responses (124). The transcription factor *Foxp3* is key for all Treg to acquire their immunosuppressive phenotype and function. Its expression is regulated epigenetically (125). Foxp3 associates with HAT (p300 or TIP60) (126) as well as HDAC (SIRT1 or HDAC5) (127) pointing HAT inhibitors (HATi) as potential drugs for inhibiting Treg function (128). In Treg, replacement of a repressor complex at the Foxp3 promoter by the HAT p300/CREB-binding protein-associated factor (PCAF) is key to enable permissive histone modifications and as such making the Foxp3 promoter accessible (129). The *Foxp3* gene also has three conserved, epigenetically regulated, noncoding regulatory sequences (CNS1-3) that modulate its expression. The first sequence (CNS1) is a TGFβ-sensitive enhancer element that is regulated *via* histone modifications and that is critical for Treg generation (125, 130). The second sequence (CNS2) is a demethylated CpG rich region, which is maintained by TET2 and allows stable *Foxp3* expression. This noncoding sequence is further characterized by H3K4 methylation, and H3 and H4 lysine acetylation (131). The importance of this region for Foxp3 expression is shown by the loss of Foxp3 expression in Treg that do no longer contain this noncoding sequence and that are exposed to IL4 and IL6 (132). The third sequence (CNS3) has been shown to be important to initiate Foxp3 expression, however, does not seem to play a role in maintaining Foxp3 expression. This sequence is rich in permissive H3K4me1/2 marks, which are highly present in nTreg, suggesting that this sequence facilitates opening the Foxp3 locus in CD4^+^ T cells that are developing in the thymus (**Figure 4**) (125).

DNA methylation is also implied in Treg development, as evidenced by methylated-DNA immunoprecipitation sequencing to assess the genome‐wide 5‐methylcytosine status. it has been estimated that 0.19% of whole genome DNA methylation sites of the conventional T cells are specifically demethylated in Treg (133). These demethylated regions are stable and occur in Treg function‐associated genes such as *Foxp3*, *Ctla4*, *Il2ra*, *Tnfrsf18* (encoding GITR), *Ikzf2* (encoding Helios), and *Ikzf4* (encoding Eos) (125, 134). Thus, it is clear that Treg and conventional T cell subsets have their own genome DNA methylation status (123). It has been reported that DNMT1 is necessary for maintenance of the Treg development program and function, showing that its deletion within the Treg lineage leads to lethal autoimmunity (135). While some authors have found that DNMT1 inhibitors could improve Treg proliferation, Treg suppressor (136–138) or Foxp3 expression (139, 140) by DNMT1 inhibitors, others reported an inhibition of Treg function (141).

Thus, overall data support a central role for epigenetic events in controlling CD4^+^ T cell fate. As the specific epigenetic drivers and pathways required for effector function and memory generation are highly context-dependent and vary with each subpopulation and location, which would be the most suitable targets to favor anti-tumoral CD4^+^ T cell responses remains to be defined. Among others, HDAC1 and HDAC11 appear potential targets for pharmacological inhibition to improve T_H_1 CD4^+^ T cell effector function. Nevertheless, many questions still remain given the complexity of the TME.

#### Epigenetics in CD8 T^+^ Cell Differentiation

##### Histone Modification and DNA Methylation in Effector and Memory Formation

**Figure 5:**
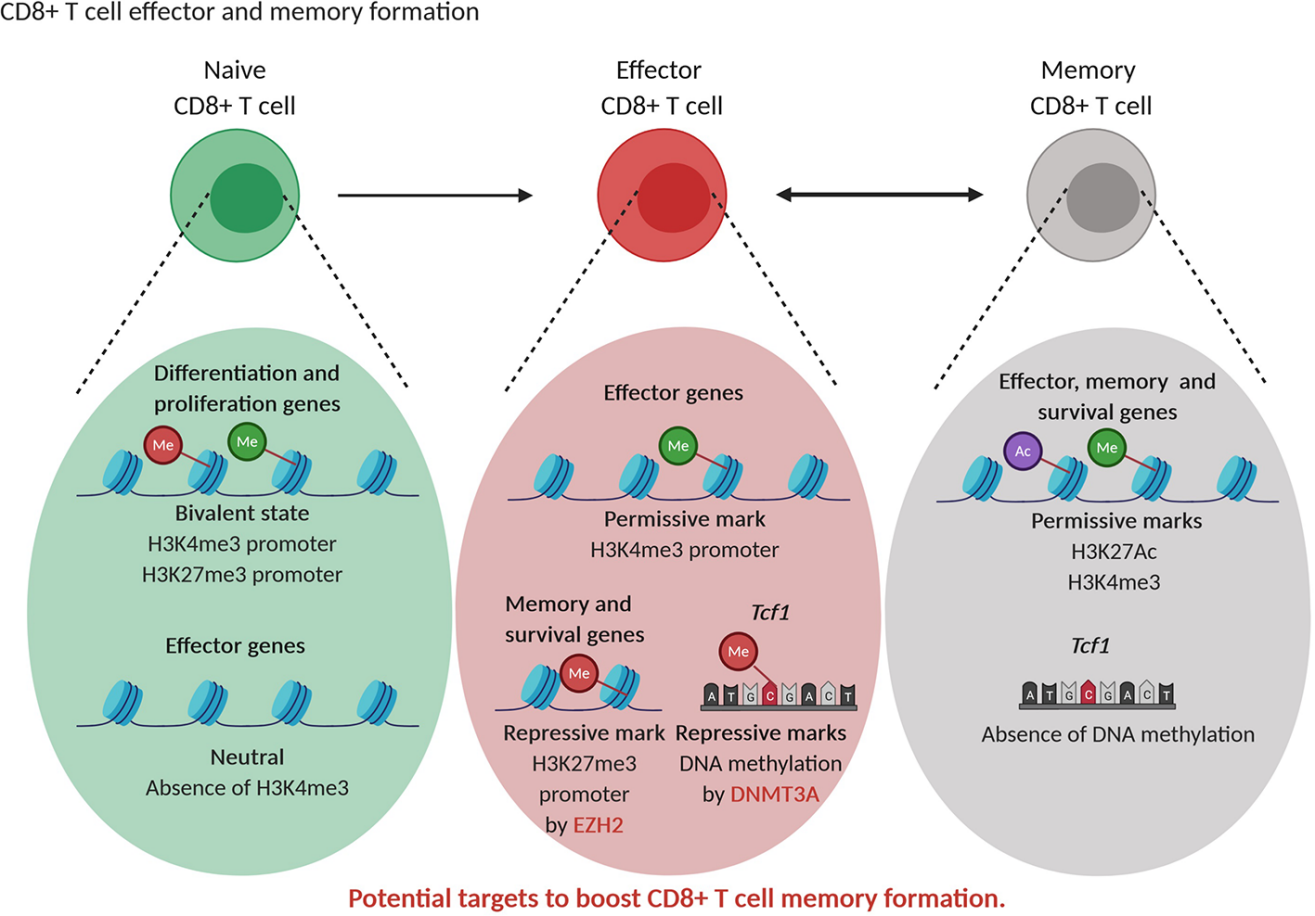
Epigenetic regulation of CD8^+^ T cell differentiation. Naive CD8^+^T cells are characterized by a bivalent state of permissive (green) and repressive (red) histone marks in genes related to differentiation and proliferation while effector genes are not decorated by permissive marks or repressive marks. Effector CD8^+^T cells show permissive (green) marks in effector genes while the transcription factor Tcf1 and memory and effector genes contain repressive (red) marks. Memory CD8^+^T cells show permissive (green, purple) marks in effector, memory and survival genes while Tcf1 is not DNA methylated, supporting their multipotent state. EZH2 and DNMT3A represent potential targets to increase CD8^+^ T memory formation.

As in the case of CD4^+^ T cells, also the differentiation of CD8^+^ T cells is characterized by alteration in histone marks and DNA modifications in effector and memory subtype specific genes (142). Whether memory CD8^+^ T cells differentiate in linear or circular fashion is still under debate. The epigenetic changes during differentiation from naive to stem cell memory, central memory and effective memory are progressive and suggest a linear model of differentiation (143, 144). As an alternative to the linear model, a circular model of differentiation is proposed where memory T cells are formed after dedifferentiation of effector T cells. This dedifferentiation is the results of demethylation of *de novo* DNA methylated genes during differentiation leading to re-expression of naive-related genes in memory cells (145). In line, murine naive T cells are characterized by the presence of bivalent state, with both repressive H3K27me3 and activating H3K4me3 marks, in genes linked to replication (“stemness”) and cellular differentiation. When naive cells start to differentiate, these loci loose bivalency as H3K27me3 is erased, allowing T cell expansion and differentiation. The H3K4me3 mark in genes related to the immune effector function, such as *Gzmb* and *Ifng* is absent in naive T cells and is acquired in effector and memory CD8^+^ T cells, thereby stimulating effector functions (144, 146). When comparing murine memory and effector CD8^+^ T cells, Gray et al. found that EZH2, through H3K27me3 deposition, repressed memory and survival genes in terminally differentiated effector cells. However, H3K27Ac mark associated with transcriptional activation was present at memory-related and effector-related loci in memory cells reflecting their multipotency (“stemness”) and allowing a fast response upon second antigen encounter (147). In addition, transition of early effector T cells into terminal effector or memory CD8^+^ T cells is dependent on the action of DNMT3A that silences the “stemness” transcription factor *Tcf1* expression through DNA methylation (**Figure 5**) (148). Knockout of DNMT3A resulted in higher numbers of memory T cells at the expense of terminal effector T cells (148).

##### Histone Modifications and DNA Methylation in Exhaustion

**Figure 6:**
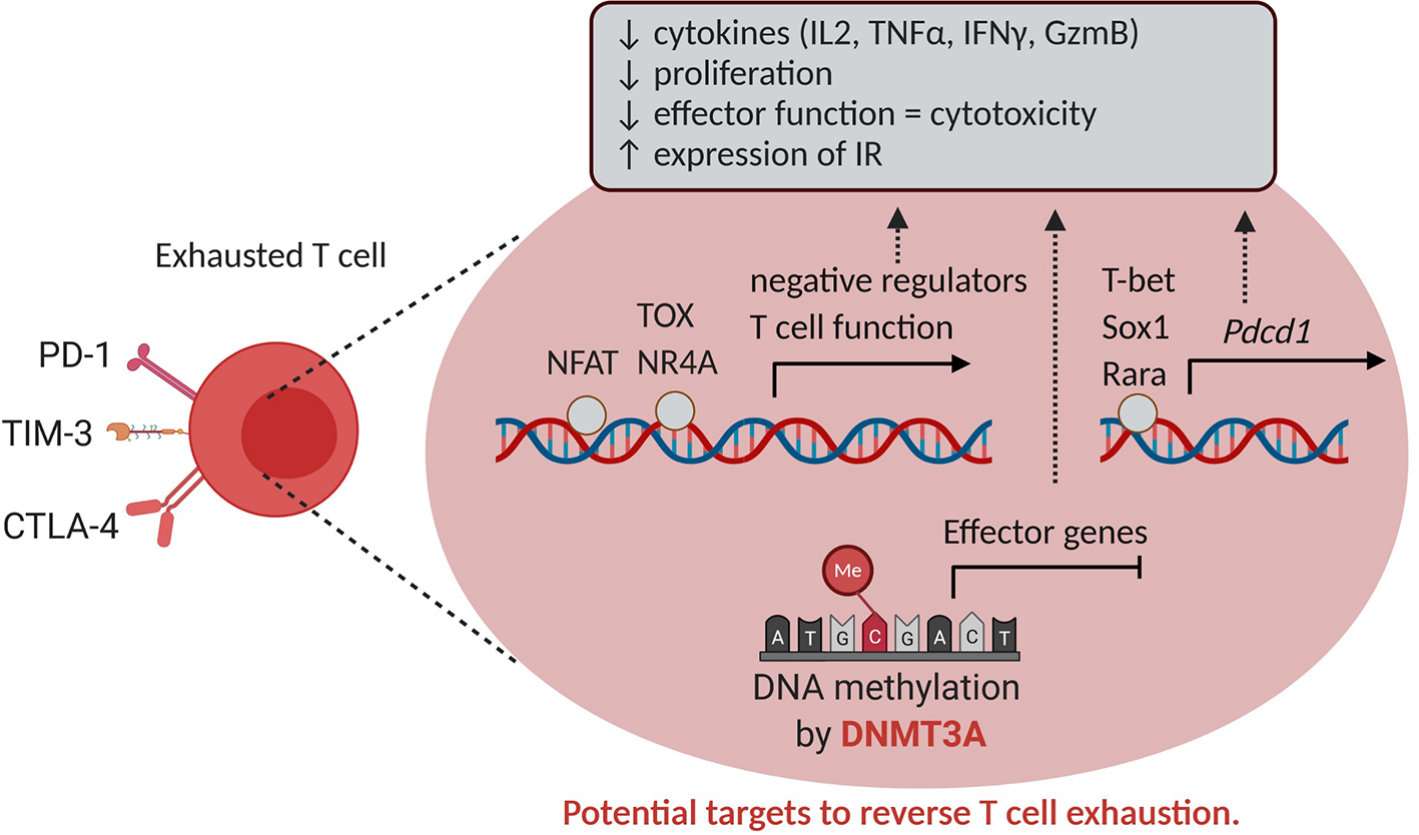
Epigenetics and T cell exhaustion. T cell exhaustion is characterized by reduced T cell proliferation, effector functions and increased expression of inhibitory receptors such as PD-1, CTLA-4 and TIM-3. The expression of these inhibitory receptors in CD8^+^ tumor-infiltrating cells is enabled altered chromatin accessibility and binding of transcription factors such as NFAT, TOX, NR4A, T-bet, Sox1 and Rara in genes exerting a negative role for T function. In addition, DNMT3A mediates DNA methylation and silencing of effector genes, hence serving as a potential target to reverse T cell exhaustion. IR, inhibitory receptor.

Epigenetic mechanisms are also involved in T cell exhaustion (**Figure 6**). Exhausted T cells are hallmarked by the expression of inhibitory receptors such as programmed death-1 (PD-1) and lack effector function, which contributes to cancer cell immune evasion. In the case of chronic viral infection, a complex gene expression program associated with broad remodeling of chromatin accessibility and enhancer landscape was described in exhausted T cells compared to functional memory and effector T cells (149). Indeed, ATAC-sequencing of acute versus chronically virus exposed murine T cells demonstrated in the latter an increased accessibility of genes associated with negative regulation of cytokine production, NF-κβ signaling, and T cell activation. Amongst those genes, *Pdcd-1*, *Havcr2* and *Batf* (encoding Pd-1, Tim3 and Batf, respectively) were identified as most likely to be bound by the transcription factors T-bet, Rara and Sox3 (149).

Also in cancer settings, epigenetic modifications progressively define unresponsive T cell phenotypes. In a murine melanoma tumor model, ATAC-sequencing similarly demonstrated in exhausted T cells an enrichment of chromatin accessibility for Nr4a and NFAT transcription factors. Of note, blocking the PD-1/PD-L1 interaction did not restore the chromatin landscape within exhausted T cells (150), as also found in a model of chronic infection, due to the persistence of an epigenetic state of exhaustion (151). Again by ATAC-Seq, it was found that tumor-specific T cells in pre-malignant lesions initially acquire a plastic dysfunctional state from which T cells can be rescued, and transition to a fixed dysfunctional chromatin state in which the cells appear resistant to reprogramming (152). Recently, the HMG transcription factor TOX was identified as a key driver of the epigenetic changes seen in exhausted T cells as a result of chronic TCR stimulation and calcineurin-NFAT2 activity (153). Of interest, *de novo* DNA methylation by DNMT3A also contributes to T cell exhaustion by repressing effector T cell genes during chronic T cell stimulation (154) (**Figure 6**).

How current therapeutic strategies impact on CD8 T cell-restricted epigenetic changes remains to date only partially understood, and yet EMA bear the potential to promote protective anti-tumor responses. Again, choosing the most appropriate target would require more extensive work. Based on the above reviewed evidences, EZH2 and DNMT3A inhibition appear a promising strategy suitable to shape effector and exhausted phenotypes of CD8^+^ T cells within the TME.

### Epigenetic Regulation of NK Cells Function in Cancer

NK cells are large granular lymphocytes and play a crucial role in the innate immune system. They develop mainly in lymphoid organs such as the bone marrow, spleen and lymph nodes. NK cells have cytotoxic and cytokine producing properties and aid in the control of tumor progression and infections. The activity of NK cells is dependent on the expression of activating and inhibitor receptors and respective ligands on the target cells. NK cells will become activated upon contact with target cells that have lost expression of ligands for inhibitory receptors (such as MHC-I) and gained stress-associated molecules that stimulate activating receptors such as NKG2D, NCR1/2/3 and Ly49D/H. As a result, NK execute their effector functions including production of cytokines such as IFNγ and direct killing of target cells (155, 156). A limited number of studies have shown that NK cell differentiation and function are regulated at the epigenetic level (157), of which some example are described below.

Yin et al. identified an important negative role for EZH2 in the development of NK cells. Knockout of EZH2 or pharmacological inhibition using UNC-1999 resulted in enhanced NK cell development from progenitors. These NK cells also showed enhanced functionality against tumor cells (158). Moreover, using a combination of microarray and ChIP-sequencing analysis, Li et al. found that NK cell activation of the NK-92MI cell line is characterized by dynamic changes in the expression of HDM and HMT as well as changes in H3K27me3 and H3K4me3 and specific loci. Inhibition of H3K27me3 using UNC-1999 resulted in increased degranulation capacity of NK cells (159).

The role of histone acetylation has also been studied in several studies, mostly by looking at the effect of HDAC inhibitors. In general, HDAC inhibitors have been found to upregulate NK activation receptor NKG2D on NK cells as well its ligand MHC class I–related genes (MIC) on tumor cells resulting in enhanced NK-cell mediated recognition and killing in hematological and solid tumors (160–162).

With respect to DNA methylation, it has been demonstrated that expression of the inhibitory Killer immunoglobulin-like receptors (KIR) is regulated by DNA methylation. The NK-92MI cell line is characterized by DNA methylation dependent repression of KIR receptors. Upon treatment with azacytidine, KIR expression is upregulated, resulting in diminished cytolytic activity of NK cells towards leukemic cells (163).

## Epigenetic Targeted Therapy Influences Tumor Immunogenicity and Immune Cell Infiltration

The development of cancer is hallmarked by immunoediting. Given that many of the events that control the process of immunoediting are regulated by epigenetic mechanisms, EMAs bear the potential to interfere with its course. The concept of immunoediting describes how the host’s immune system interacts with a developing tumor in three consecutive phases. In the elimination and equilibrium phases, the immune system successfully removes or controls the growth of the tumor allowing the recovery of the normal tissue architecture. In the escape phase, however, tumor cells selected during the equilibrium phase are able to grow out in an immunocompetent setting due to several immune evasion mechanisms which essentially can influence each step of the cancer-immunity cycle (164). First, low amounts of tumor antigens, poor antigen presentation and failure in undergoing immunogenic cell death (ICD) limits priming of effector immune responses and induces immune ignorance. Second, poor infiltration of effector immune cells, tolerant phenotypes of DC and macrophage, and the presence of immune suppressive immune cells such as Treg and MDSC limit the priming and execution phase of the anti-tumor immune responses. Third, the function of immune effector cells can be inhibited by the presence of checkpoint molecules and absence of costimulatory molecules (165, 166). As a consequence, tumors can be classified based on their baseline level of immunity: (i) “immune cold” tumors that lack antigen presentation, have low adjuvanticity and hence have poor priming of immune response and lack functional anti-tumor T cells; (ii) “immune intermediate” tumors with proper immune cell priming but where immune responses are hindered by a multitude of mechanisms including distorted chemokine signaling, poor CD8^+^ T cell infiltration, hypoxia, tumor cell intrinsic factors and presence of immunosuppressive molecules and cell types (MDSC, M2 macrophages, Treg); and (iii) “immune hot” tumors that show good levels of immune priming and CD8^+^ T cell infiltration but show exhaustion (167, 168).

**Figure 7:**
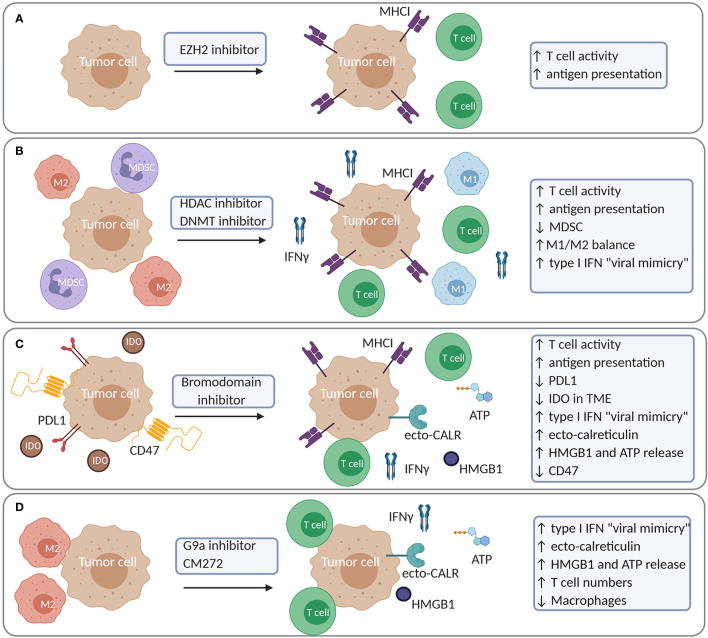
Examples of increased tumor immunogenicity and modulation of the immune cell constitution in the TME upon treatment with epigenetic compounds. **(A)** EZH2 inhibition results in increased antigen presentation and T cell activity. **(B)** HDAC or DNMT inhibition increases antigen presentation and T cell activity while reducing MDSC and M2 macrophages. **(C)** Bromodomain inhibitors (JQ1) increase antigen presentation and T cell activity while it reduces tumoral PDL1 expression and IDO in the TME. JQ1 also induces a more complete picture of ICD with a type I IFN response, ecto-calreticulin, HMGB1 and ATP release. JQ1 moreover reduces CD47 expression in tumor cells **(D)** G9a inhibitors and CM-272 induce a type I IFN response due to viral mimicry in tumor cells. CM-272 induces a more complete picture of ICD with a type I IFN response, ecto-calreticulin, HMGB1 and ATP release. IDO, Indoleamine 2,3-dioxygenase.

Recently, Burr et al. have identified that the polycomb repressor complex 2 (PCR2), which includes EZH2, drives immune evasion through epigenetic silencing of genes involved in antigen presentation through MHC-I. Epigenetic silencing was mediated through deposition of H3K27me3 in the promoter region of these genes, leading to reduced expression, even in response to cytokines such as IFNγ (169). Of interest, blocking the PRC2 function through inhibition of EZH2 methyltransferase activity restored antigen presentation at basal level and enhanced antigen presentation after cytokine stimulation in multiple tumor models. Consequently, the anti-tumor activity of T cells was restored in tumors treated with the EZH2 inhibitor (169). Similar findings were observed in diffuse large B cell lymphoma (DLBCL) where EZH2 inhibition restored MHC expression in DLBCL cell lines (170). EZH2 was also shown to be upregulated in response to TNFα induced by CTLA-4 blockade and IL2 agonist treatment in B16 and RIM3 melanoma models (171). This resulted in the downregulation of tumor antigen presentation and acquired resistance to immunotherapy. Of note, blocking EZH2 reversed these effects and restored T cell activity (171). Importantly, EZH2 and its inhibition might have different effects according to the tumor type and the immune infiltrate. For example, in a mesothelioma model, EZH2 regulated macrophage-induced oxeiptosis of mesothelioma cells, and its blocking impaired macrophage function and tumor control (172). These findings support a positive role for EZH2 in macrophage function, also found in inflammatory models (173, 174). Thus, although EZH2 might be a good target to restore tumor cell antigenicity and immunogenicity (**Figure 7A**), pleiotropic effects should be studies, due to the various functions EZH2 might play with TME immune cell subsets.

In addition to histone methylation, a number of studies support the notion that the targeting histone (de)acetylation and DNA methylation enhances tumor recognition by immune cells (**Figure 7B**). In multiple tumor cell lines, the HDACi Trichostatin A induced the expression of genes involved in antigen presentation (175, 176), which facilitated tumor cell killing by CTL (176). Similar findings were observed in a melanoma model, where Trichostatin A induced MHC-I expression promoting IFNγ production by T cells specific for a tumor-associated antigen (177). Also in the case of chronic lymphocytic leukemia (CLL), the expression of antigens, costimulatory genes and T cell responses was increased after Decitabine and HDACi LAQ824 treatment (178). In breast and prostate cancer cells, the pan-HDACi Vorinostat and the HDAC class I-specific inhibitor Entinostat similarly favored the expression of components of the antigen-presentation machinery and augmented T cell-mediated cell lysis (179). The combination of Decitabine and HDACi Valproic Acid resulted in augmented antigen expression in mesothelioma cells resulting in increased T cell recognition and killing capacity (180). Moreover, in a model for breast cancer, the HDAC class IIA inhibitor TMP195 increased the presence of highly phagocytic macrophages in the TME leading to anti-tumor effects and reduced metastasis (181). In lung cancer, combining HDACi with DNA demethylating agents reduced macrophage infiltration, increased T cell infiltration of the TME with non-exhausted T cells and reduced MYC expression in tumor cells (10). Similar findings have been observed in multiple myeloma, where the combination of Decitabine and HDACi Quisinostat reduced MDSC and induced temporal changes in memory T cells in the bone marrow while reducing MYC expression in tumor cells (11).

In several tumor models, BET-bromodomain inhibitors such as JQ1 also restored tumor immunogenicity by increasing MHC-I and reducing PD-L1 expression in tumor cells leading to their enhanced recognition by T cells (182–184) (**Figure 7C**). In a melanoma model, a new BET inhibitor, PLX51107, reduced tumor growth by increasing T cell activity and lowering PD-L1 and IDO expression in the TME (185). In non-small cell lung cancer, a combination of a HDAC6 inhibitor Riclinostat and JQ1 reduced tumor growth though diminishing Treg and increasing T cell and DC activity (186).

The use of epigenetic therapy as ICD inducer has gained attention the last few years (187). During ICD, tumor cells emit, in a spatiotemporal manner, several danger-associated molecular patterns (DAMPs) that can trigger an anti-tumor adaptive immune response. Evidence that epigenetic-treated cells can serve as a vaccine has been shown by Khang et al. Vaccination of mice with Trichostatin-A treated cells resulted in a delay in tumor development in melanoma and plasmacytoma (188, 189). Our group has investigated whether epigenetic-targeted therapy could trigger ICD in multiple myeloma using the 5T33 model (11). In this model, combined use of Decitabine and the HDACi Quisinostat could not trigger *bona fide* ICD. This was based on the observation that vaccination with treated tumor cells only delayed tumor development but did not lead to complete protection after challenge with living tumor cells. Ecto-calreticulin expression was induced to some extent but the “don’t eat me” signal CD47 was also present, possibly counteracting the effects of ecto-calreticulin. Nevertheless, a robust type I IFN response was induced, which potentially contributed to the observed DC activation (11). The induction of type I IFN has been reported in the context of DNA demethylating agents as being the result of the induction of endogenous retroviruses (“viral mimicry”) and activation of endogenous dsRNA sensors MDA5, RIG1 and TLR3 leading to a type I IFN response (190, 191). Also combinations of G9a and DNMT inhibitors strongly induced viral mimicry in ovarian cancer and hematological malignancies (8, 12). Recent work by Jung et al. identified that silencing of genes involved in type I IFN gene signaling and dsRNA sensor pathways could hamper a “viral mimicry” response in tumors characterized by high mutational load and copy number changes, leading to immune evasion and immunotherapy resistance. Such tumors may be of particular interest to test whether epigenetic drugs could restore the “viral mimicry” response (192). With respect to the emission of DAMPs, *in vitro* and *in vivo* HMGB1 release in several tumor cell lines has been observed in response to the DNA demethylating agents Decitabine and Azacytidine as well as the HDACi Vorinostat (193). Calreticulin exposure has also been observed in childhood brain tumors upon treatment with Vorinostat (194). A more complete picture of ICD hallmarks was observed using CM-272, a dual G9a/DNMT1 inhibitor, developed by José-Eniréz et al. (12, 13). In bladder cancer, CM-272 induced viral mimicry, a type I IFN response, ecto-calreticulin and HMGB1 release. This was accompanied by an increase in CD8^+^ T cells and NK cells, and a decrease in macrophages (13). The BET-bromodomain inhibitor, JQ1, induced ecto-calreticulin, HMGB1 and ATP release in oral squamous carcinoma. In this model, vaccination with JQ1-treated cells conferred protection upon a subsequent challenge with living cells (195). Also in breast cancer, JQ1 has been reported to decrease the expression of CD47, through disruption of the super enhancers that regulate CD47 expression (196). It is clear that epigenetic-targeted therapy has the potential to restore several mechanisms of immune evasion. There is increased tumor cell immunogenicity and multiple studies also support the induction of ICD, depending on the tumor model (**Figures 7A-D**). In addition, epigenetic treatment modulates the immune cell constitution of the TME, thus bearing the potential to reprogram an immunosuppressive environment into a favorable one.

## Epigenetic-Targeted Therapy Shows Promise in Combination With Immunotherapy

Multiple mechanisms of anti-tumor immunity can be identified in response to epigenetic-targeted therapy and depending on the underlying mechanisms, combinations with other immunotherapeutic strategies can be applied to obtain synergistic effects.

### Epigenetic Treatment and Vaccination

A few studies have investigated the combination of epigenetic therapies with vaccination strategies. Here, the rationale of using the epigenetic compounds is primarily to induce the expression and presentation of antigens on tumor cells. A phase I study in myelodysplastic syndrome (MDS) tested the combination of NY-ESO-1 vaccination (CDX-1401) and Decitabine. CDX-1401 is a DEC-205/NY-ESO-1 fusion protein and was given together with the TLR3 agonist Poly-ICLC, as an adjuvant. The therapy led to induction of NY-ESO-1 expression (by Decitabine) and NY-ESO-1 specific CD4^+^ and CD8^+^ T cell responses, which was associated with induction of CD141^+^ DC (197).

Similar findings were observed in ovarian cancer where Decitabine induced NY-ESO-1 expression and anti-NY-ESO-1 specific antibodies in combination with NY-ESO-1 protein vaccine and Doxorubicin (198). In childhood neuroblastoma and sarcoma, Decitabine has been tested together with a DC vaccine pulsed with MAGE-A1, MAGE-A3 and NY-ESO-1 peptides (199). In all three studies, responses were heterogeneous and short-term. Yong Lee et al. tested the combination of the pan-HDACi AR-42 together with the DNA vaccine encoding for calreticulin and human papilloma virus protein E7 (CRT/E7) in a TC-1 lung cancer model. Addition of AR-42 enhanced the effects of the vaccine and induced CD8^+^ T cell responses leading to better anti-tumor responses compared to vaccination alone (200). More recently, in the B16-OVA melanoma model, a combination of the HDACi Romidpesin and the BET-inhibitor IBET-151 together with adenoviral- or protein-based vaccines were tested. Epigenetic treatment resulted in increased numbers of antigen specific CD8^+^ T cells and a better therapeutic outcome (201).

### Epigenetic Treatment and Checkpoint Blockers

Combinations of epigenetic therapies with checkpoint blockers are the most studied and most promising treatment at the moment. Recently, modulation of histone methylation has been proven to enhance checkpoint inhibition. EZH2 inhibition together with anti-CTLA-4 has combinatory effects in melanoma and bladder cancer models. These effects could be linked to decreased number of Treg and increased numbers of CD8^+^ T cells (202). In breast cancer, the use of a lysine demethylase 1 (LSD1) inhibitor led to increased chemokine expression, T cell infiltration and combinatory effects with anti-PD-1 blockade (203). Of specific interest, the dual G9a/DNMT1 inhibitor CM-272 showed synergy with anti-PD-L1 blocking therapy in metastatic bladder cancer, which is in line with the strong ICD-inducing properties of CM-272 (13).

Also HDACi have been tested in combination with checkpoint blockers. Woods et al. showed that the pan-HDACi Panobinostat induced PD-L1 and PD-L2 in human and mouse melanoma cells through increased lysine acetylation of their promoter. In an *in vivo* experiment, combinations of PD-1 blocking and Panobinostat resulted in a significant delay of survival compared to single agent treatment (204). Similar findings were observed in human and murine lymphoma cells with the use of Class I HDACi (valproic acid), HDAC1/2 (Romidepsin) and HDAC3 inhibitors (RGFP966) (205). In a syngeneic murine lymphoma model, RGFP966 induced PD-L1 in tumor cells and DC and synergized with anti-PD-L1 blocking (205). In ovarian cancer, the SWI/SNF chromatin remodeling complex ARID1A is mutated in >50% of the cases leading to increased PD-L1 expression. Hence, blocking PD-L1 together with the HDAC6 inhibitor ACY-1215 showed combinatory anti-tumor effects which were dependent on CD8^+^ T cells (206). In hepatocellular carcinoma, combined anti-CTLA-4 and PD-1 together with the HDACi Belinostat significantly reduced tumor burden; which was associated with increased IFNγ production by T cells, and decreased amounts of Treg (207). Several other reports also showed that HDACi such as Entinostat and Mocetinostat synergized with checkpoint inhibition by targeting immature myeloid cells in solid tumor models (96, 97, 208). In lung adenocarcinoma, Zheng et al. identified that HDACi induced chemokine expression in tumor and surrounding cells leading to T cell attraction and increased sensitivity to anti-PD-1 therapy (209). Of interest, in multiple carcinoma models, Hicks et al. have found that HDAC inhibitors could upregulate NK cell ligands and death receptors leading to enhanced killing of tumor cells by NK-cells. At the same time, anti-PD-L1 was upregulated in tumor cells leading to antibody-dependent cellular toxicity using avelumab (210).

DNA methyltransferase inhibitors have also been tested in several cancer models. The combination of Azacytidine and CTLA-4 blocking antibodies showed combinatory effects in the B16-F10 melanoma model (190). This is thought to be the result of a type I IFN response that was induced by Azacytidine as the presence of this type I IFN gene signature is predictive for response to CTLA-4 blockade in melanoma patients (190). In murine lung cancer, Decitabine led to demethylation of *Irf7* and induced the expression of PD-L1, type I IF and chemokines such as CXCL9 and CXCL10 leading to sensitization of tumors to anti-PD-1 blockade (211). Similar findings were found in breast cancer models, where Guadecitabine induced MHC-I expression and NF-κβ and IFN signaling, resulting in combinatory effects with anti-PD-L1 blockade (212). In the Tramp-C2 model, which appeared to be resistant to PD-L1 blockade, Decitabine pre-treatment could restore sensitivity towards anti-PD-L1 as a result of reprogramming exhausted T cells (154). HDACi and DNMTi combination also appear to synergize with checkpoint blockers, which has been demonstrated in ovarian, colorectal and lung cancer (9, 213). Moreover, this combination also significantly altered the immune cell constitution in the TME by reducing macrophages, MDSC and increasing immune-effector cells (9, 213). Taken together, epigenetic treatment can synergize with immune checkpoint inhibition through direct regulation of immune checkpoint expression, modulation of T cell infiltration and reduction of immature myeloid cell infiltration.

### Epigenetic Treatment and Adoptive Cell Therapy Including CAR-Therapy

Adoptive T cell therapy (ACT) is a passive immunotherapeutic approach in which antigen specific T cells are delivered to patients to elicit CTL responses. Targeting of DNA methylation is of interest to augment the T cell efficacy during ACT. Indeed, Decitabine has been shown to increase expression of P1A antigen in several solid and hematological tumor models. Hence, adoptive transfer of P1A specific T cells resulted in significant improvement of anti-tumor CTL responses (214). In a phase II clinical trial in multiple myeloma, autologous T cell infusion was combined with Lenalidomide and Azacytidine. Following Lenalidomide and Azacytidine treatment, patients underwent autologous stem cell transplantation followed by autologous T cell infusion (215). The treatment resulted in upregulation of cancer-testis antigens in multiple myeloma cells with some evidence of ongoing antigen specific CTL responses (215). In the murine 4T1 breast cancer model, Decitabine enhanced the anti-tumor efficacy of ACT together with Cyclophosphamide. This was associated with increased MHC-I and antigen expression and decreased number of MDSC in the TME (216). In the B16 melanoma model, the pan-HDACi LAQ824 and Panobinostat has been shown to boost activity of gp100 specific T cell responses (217, 218). Mechanistically, LAQ824 increased the activity of the T cells and induced antigen expression (218). Moreover, Panobinostat induced a pro-inflammatory environment and induced CD25 and OX40 expression in T cells (217). Targeting DNMTs by Decitabine, together with inhibition of EZH2, significantly increased antigen expression in human lung cancer cells. This led to increased antigen specific CTL responses (219). Of note, EMA might also overcome acquired resistance consequent to ACT. In preclinical mouse models, the treatment with EMAs, and in particular DNMTi, was effective in reinstating the expression of antigens silenced as a consequence of immunoediting. The combination of EZH2 and DNMT1 inhibitors in ovarian cancer also promoted the intratumoral expression of T_H_1 chemokines (CXCL9 and CXCL10) expression, which correlated with improved efficacy of adoptive T cell transfer and checkpoint inhibition (220).

EMA can also be exploited during the production of a T cell product, including CAR-T cells. Kagoya et al. tested the effects of the BET-bromodomain inhibitor JQ1 during the cultivation and *in vitro* expansion of T cells for adoptive transfer. JQ1 treatment led to T cells with increased stem cell and memory-like properties by regulating BTAF expression. These T cells showed enhanced capacity to control tumor growth in leukemia and melanoma models (221). Likewise, the accidental disruption of the enzyme TET2 during the generation of CD19 specific CAR-T cells resulted unexpectedly in improved therapeutic efficacy in chronic lymphocytic leukemia. The disruption of TET2 resulted in an altered T cell differentiation with a predominance of central memory T cells, thereby improving the efficacy and persistence of CAR-T cells. The authors further showed that similar results might be reached by TET2 inhibition during the production phase (222). These findings suggest that the balance of DNA methylation events in effector genes and memory (“stemness”) genes during the transition of early to terminal effector, memory or exhausted T cells, eventually determines the highest functionality of T cells which is thought to be reached in the functional memory state (223). EMAs have also been shown to synergize with CAR-T cells. For instance, Decitabine treatment of lymphoma cells reduced DNA methylation, thereby improving CD19 expression and favoring recognition by CD19 specific CAR-T cells both in patients with B-cell lymphoma and *in vitro* cultures (224). In Ewing sarcoma, inhibition of EZH2 has demonstrated to promote anti-tumor activity of G_D2_ specific CAR-T cells and more efficient tumor cell lysis (225). In summary, epigenetic therapy can improve the outcome of ACT, by mediating the expression of antigens and improving the activity and attraction of T cells. Of note, the efficacy of engineered tumor-targeted T cells relies on the contribution of endogenous T cells, which limit immune escape upon ACT (226–228). Whether EMAs would further improve host T cell immunity in the context of TCR or CAR-T cell therapy, by promoting better antigen presentation, and better tumor infiltration by polyclonal T cells remains to be understood. In addition, whether improved responsiveness to ACT depends on reprogramming of additional immune cell subsets represented within the TME remains an important open question.

In addition to T cells, also NKs are promising candidate for CAR-engineering. They represent 10–20% of human peripheral blood leukocytes, and as such an attractive source for genetically modified immune cell-based immunotherapy. These cells of the innate immune system target cancer cells that down-regulate HLA class I molecules and have the advantage that they can be derived from HLA mismatched donors. The use of CAR-engineered NK cells has been proven in preclinical mouse models and in phase I/II clinical trials (157, 229). As discussed in previous paragraphs, epigenetic mechanisms including histone- and DNA methylation-based modifications regulate the activity of NK cells, and also the ability of cancer cells to evade NK cells. Thus, combination of NK cell-based immunotherapy with epigenetic therapies is likely to enhance efficacy (157). To date, clinically approved EZH2, IDH1, and IDH2 inhibitors are being tested in immunocompromised preclinical models of cancer. In addition, EZH2 and HDAC inhibitors could also be exploited during the generation of CAR-NK cells to overcome difficulties in the expansion and genetic manipulation of NK cells (156) and promote survival and functionality of engineered NK cells (158, 161).

## Conclusion and Future Perspectives

Immunotherapy has emerged as the fourth pillar of anti-cancer therapy with encouraging results in clinical trials on cancer vaccination, CAR-T cell and immune checkpoint therapy (230–234). However, the occurrence of immune resistance or side effects emphasizes the need to identify therapy modalities that can be combined with immunotherapy to fully capitalize on the immune system’s ability to eradicate tumor cells.

Epigenetics, which is a reversible process, regulate the phenotype of cancer cells as well as of immune cells and have therefore been proposed as a common denominator for modulation. The discovery of modifiable epigenetic pathways that can shape the immune response has opened novel therapeutic perspectives, with the potential of improving current strategies. Indeed, epigenetic therapy has the ability to modulate the TME in various ways, for example, by (i) enhancing the immunogenicity of tumor cells, (ii) inducing accumulation and infiltration of CD8^+^ CTL and linked herewith (iii) preventing acquisition of an exhausted state. Moreover, epigenetic therapy can affect the myeloid cell compartment, jumpstarting antigen presentation to T cells by DC, while curtailing immunosuppressive myeloid cell types such as M2 polarized TAM and MDSC.

Nonetheless, several challenges remain, these can be summarized as (i) designing selective EMAs with optimal pharmacokinetics, target inhibition, safety, bio-distribution, and efficacy (ii); fully understanding their activity in the TME, and linked herewith (iii) defining biomarkers that help to identify patients eligible for the therapy and to monitor therapy response. Although several epigenetic mechanisms have been identified in immune cells as reviewed here, efforts to improve our understanding of the epigenome of immune cells in the context of cancer is necessary. The fact that targeting epigenetic processes might yield counterbalancing effects on different immune cells makes it difficult to predict upfront the final outcome of the given treatment. Targeting of EZH2 might represent a good example of this notion. Indeed, EZH2 has generally been considered an interesting target to improve anti-tumor T cell responses, as its inhibition promotes T_H_1 differentiation, CD8^+^ T cell memory formation and increases tumor cell immunogenicity. However, inhibiting EZH2 might concomitantly promote to increased MDSC differentiation, which may counteract the positive effects on anti-tumor immunity. The same holds true for G9a, as its inhibition improves tumor immunogenicity, promotes M1 polarization and DC function, but may also promote Treg development. Consequently, to fully understand the putative impact of EMAs, it will be crucial to precisely define their effects on tumor types and on the various TME components, and use representative, immunocompetent mouse models. We believe that orthotopic or genetically engineered rodent models in which the tumor is developing in its primary organ or *ex vivo* organoids with human tumor and immune cells are most suitable for this purpose. To guide the selection of EMAs, or a combination thereof, it is crucial to understand the baseline immunity status of the tumor by examining its immune cell constitution in addition to the functional state of immune cells. This can be achieved using multiparametric flow cytometry analysis and/or immunohistochemistry analyses of the TME coupled to single cell sequencing suitable to unveil epigenetic and transcriptomic signatures. Said that, “immune cold” tumors would benefit of strategies able to improve antigenicity and immunogenicity and efficient T cell priming while preventing T cell exhaustion and general immunosuppression over the course of the cancer-immunity cycle. “Immune intermediate” tumors would not necessarily require new priming but rather benefit of therapies that sustain effector function and inhibit immunosuppressive cell types, while “immune hot” tumors might mostly take advantage of strategies suitable to promote long-lasting and systemic memory T cell formation, and prevent or reverse exhaustion (167). EMAs might reveal beneficial at different time over the course of treatment and according to the disease state and the immunocompetence of the patient. Monitoring EMAs activity and efficacy remains challenging, although the above mentioned techniques applied to patient sample or tumor biopsies might lead to the discovery of predictive biomarkers. To this regard, the expression level of epigenetically regulated genes was used to successfully monitor pharmacodynamics effects of EMAs (235). Also, the assessment of post-translational histone modifications in clinical samples allow monitoring the response to EMAs (236). Defining the EMA-induced shaping of the immune cell representation in the TME in mouse models might instruct the use of EMAs in patients, and might also clarify the effects related to anti-tumor immunity.

The combination of EMAs and immunotherapy is emerging as a crucial therapy paradigm across a variety of cancers (237). A number of proof‐of‐concept preclinical studies combining EMAs with checkpoint inhibitors showed near complete cancer regression in tumor bearing mice, warranting further research into the subject. Also, data originated in clinical trials have highlighted the potential of EMAs for treatment of human cancers. Very recent reviews have been published elsewhere (238–240), highlighting the possibility to combine EMAs in combination with standard of care chemotherapy or radiotherapy, and also front-line targeted therapy and immunotherapy (238). In the case of immunotherapy, several combinations with HDACi and DNMTi are currently being tested in patients (238). We believe that, as strategies suitable to monitor disease state and immunocompetence in patients treated with EMAs are becoming more accessible and more widely adopted, additional information will progressively become available and guide the use of EMAs as single agents or combined with immunotherapy including cancer vaccination, immune checkpoint blockers, and TCR or CAR-T cell therapy.

## Author Contributions

KM and KB wrote the manuscript, made the figures, and critically reviewed the manuscript. AM and JJL wrote the manuscript and critically reviewed the manuscript. XA, KV, and FP critically reviewed the manuscript. All authors contributed to the article and approved the submitted version.

## Funding

KM, KV, and KB: This work was funded by FWO-Vlaanderen and Vrije Universiteit Brussel under the strategic research program scheme SRP48 and ERA-NET TRANSCAN-2 2015 (EPICA) under research grant G0H7216N and Wetenschappelijk Fonds Willy Gepts of the UZ Brussel. FP, XA, and JJL: This work was funded by Foundation for Applied Medical Research, University of Navarra (Pamplona, Spain), Fundación Fuentes Dutor, Instituto de Salud Carlos III (ISCIII) PI16/02024, PI17/00701, PI20/01306, Ministerio de Ciencia e Innovación (PID2019-108989RB-I00) and ERA-NET TRANSCAN-2 2015 AC16/00041 (EPICA), co-financed with FEDER funds), MINECO Explora SAF2017-92632-EXP (RTHALMY), Multiple Myeloma Research Foundation Networks of excellence, the International Myeloma Foundation (Brian D. Novis), and the Qatar National Research Fund award 7-916-3-237. AM: This work was funded by ERA-NET TRANSCAN-2015—EPICA and Associazione Italiana per la Ricerca sul Cancro (AIRC IG 2014 Id.15883 and 2018 Id.21763).

## Conflict of Interest

The authors declare that the research was conducted in the absence of any commercial or financial relationships that could be construed as a potential conflict of interest.

## References

[1] Berdasco M Esteller M. Aberrant epigenetic landscape in cancer: how cellular identity goes awry. Dev Cell (2010) 19(5):698–711. 10.1016/j.devcel.2010.10.005 21074720

[2] Liu M Zhou J Chen Z Cheng AS. Understanding the epigenetic regulation of tumours and their microenvironments: opportunities and problems for epigenetic therapy. J Pathol (2017) 241(1):10–24. 10.1002/path.4832 27770445

[3] Dawson MA Kouzarides T. Cancer epigenetics: from mechanism to therapy. Cell (2012) 150(1):12–27. 10.1016/j.cell.2012.06.013 22770212

[4] Bannister AJ Kouzarides T. Regulation of chromatin by histone modifications. Cell Res (2011) 21(3):381–95. 10.1038/cr.2011.22 PMC3193420 21321607

[5] Lawrence M Daujat S Schneider R. Lateral Thinking: How Histone Modifications Regulate Gene Expression. Trends Genet (2016) 32(1):42–56. 10.1016/j.tig.2015.10.007 26704082

[6] Wang D Kon N Lasso G Jiang L Leng W Zhu WG. Acetylation-regulated interaction between p53 and SET reveals a widespread regulatory mode. Nature (2016) 538(7623):118–22. 10.1038/nature19759 PMC5333498 27626385

[7] Greenberg MVC Bourc’his D. The diverse roles of DNA methylation in mammalian development and disease. Nat Rev Mol Cell Biol (2019) 20(10):590–607. 10.1038/s41580-019-0159-6 31399642

[8] Liu M Thomas SL DeWitt AK Zhou W Madaj ZB Ohtani H. Dual Inhibition of DNA and Histone Methyltransferases Increases Viral Mimicry in Ovarian Cancer Cells. Cancer Res (2018) 78(20):5754–66. 10.1158/0008-5472.CAN-17-3953 PMC6191339 30185548

[9] Stone ML Chiappinelli KB Li H Murphy LM Travers ME Topper MJ. Epigenetic therapy activates type I interferon signaling in murine ovarian cancer to reduce immunosuppression and tumor burden. Proc Natl Acad Sci USA (2017) 114(51):E10981–E90. 10.1073/pnas.1712514114 PMC5754782 29203668

[10] Topper MJ Vaz M Chiappinelli KB DeStefano Shields CE Niknafs N Yen RC. Epigenetic Therapy Ties MYC Depletion to Reversing Immune Evasion and Treating Lung Cancer. Cell (2017) 171(6):1284–300 e21. 10.1016/j.cell.2017.10.022 29195073 PMC5808406

[11] De Beck L Melhaoui S De Veirman K Menu E De Bruyne E Vanderkerken K. Epigenetic treatment of multiple myeloma mediates tumor intrinsic and extrinsic immunomodulatory effects. Oncoimmunology (2018) 7(10):1–13. 10.1080/2162402x.2018.1484981 PMC6169579 30288346

[12] San Jose-Eneriz E Agirre X Rabal O Vilas-Zornoza A Sanchez-Arias JA Miranda E. Discovery of first-in-class reversible dual small molecule inhibitors against G9a and DNMTs in hematological malignancies. Nat Commun (2017) 8:15424. 10.1038/ncomms15424 28548080 PMC5458547

[13] Segovia C San Jose-Eneriz E Munera-Maravilla E Martinez-Fernandez M Garate L Miranda E. Inhibition of a G9a/DNMT network triggers immune-mediated bladder cancer regression. Nat Med (2019) 25(7):1073–81. 10.1038/s41591-019-0499-y 31270502

[14] Lahmar Q Keirsse J Laoui D Movahedi K Van Overmeire E Van Ginderachter JA. Tissue-resident versus monocyte-derived macrophages in the tumor microenvironment. Biochim Biophys Acta (2016) 1865(1):23–34. 10.1016/j.bbcan.2015.06.009 26145884

[15] Wang N Liang H Zen K. Molecular mechanisms that influence the macrophage m1-m2 polarization balance. Front Immunol (2014) 5:614. 10.3389/fimmu.2014.00614 25506346 PMC4246889

[16] Bolli E Movahedi K Laoui D Van Ginderachter JA. Novel insights in the regulation and function of macrophages in the tumor microenvironment. Curr Opin Oncol (2017) 29(1):55–61. 10.1097/CCO.0000000000000344 27792052

[17] Awad RM De Vlaeminck Y Maebe J Goyvaerts C Breckpot K. Turn Back the TIMe: Targeting Tumor Infiltrating Myeloid Cells to Revert Cancer Progression. Front Immunol (2018) 9:1977. 10.3389/fimmu.2018.01977 30233579 PMC6127274

[18] Tikhanovich I Zhao J Olson J Adams A Taylor R Bridges B. Protein arginine methyltransferase 1 modulates innate immune responses through regulation of peroxisome proliferator-activated receptor gamma-dependent macrophage differentiation. J Biol Chem (2017) 292(17):6882–94. 10.1074/jbc.M117.778761 PMC5409459 28330868

[19] Fan Z Li J Li P Ye Q Xu H Wu X. Protein arginine methyltransferase 1 (PRMT1) represses MHC II transcription in macrophages by methylating CIITA. Sci Rep (2017) 7:40531. 10.1038/srep40531 28094290 PMC5240148

[20] Zhao J O’Neil M Vittal A Weinman SA Tikhanovich I. PRMT1-Dependent Macrophage IL-6 Production Is Required for Alcohol-Induced HCC Progression. Gene Expr (2019) 19(2):137–50. 10.3727/105221618X15372014086197 PMC6466176 30236171

[21] Kittan NA Allen RM Dhaliwal A Cavassani KA Schaller M Gallagher KA. Cytokine induced phenotypic and epigenetic signatures are key to establishing specific macrophage phenotypes. PloS One (2013) 8(10):e78045. 10.1371/journal.pone.0078045 24205083 PMC3804553

[22] Satoh T Takeuchi O Vandenbon A Yasuda K Tanaka Y Kumagai Y. The Jmjd3-Irf4 axis regulates M2 macrophage polarization and host responses against helminth infection. Nat Immunol (2010) 11(10):936–44. 10.1038/ni.1920 20729857

[23] De Santa F Narang V Yap ZH Tusi BK Burgold T Austenaa L. Jmjd3 contributes to the control of gene expression in LPS-activated macrophages. EMBO J (2009) 28(21):3341–52. 10.1038/emboj.2009.271 PMC2752025 19779457

[24] Ishii M Wen H Corsa CA Liu T Coelho AL Allen RM. Epigenetic regulation of the alternatively activated macrophage phenotype. Blood (2009) 114(15):3244–54. 10.1182/blood-2009-04-217620 PMC2759649 19567879

[25] Liu C Yu Y Liu F Wei X Wrobel JA Gunawardena HP. A chromatin activity-based chemoproteomic approach reveals a transcriptional repressome for gene-specific silencing. Nat Commun (2014) 5:5733. 10.1038/ncomms6733 25502336 PMC4360912

[26] Chen X El Gazzar M Yoza BK McCall CE. The NF-kappaB factor RelB and histone H3 lysine methyltransferase G9a directly interact to generate epigenetic silencing in endotoxin tolerance. J Biol Chem (2009) 284(41):27857–65. 10.1074/jbc.M109.000950 PMC2788836 19690169

[27] Yoshida K Maekawa T Zhu Y Renard-Guillet C Chatton B Inoue K. The transcription factor ATF7 mediates lipopolysaccharide-induced epigenetic changes in macrophages involved in innate immunological memory. Nat Immunol (2015) 16(10):1034–43. 10.1038/ni.3257 26322480

[28] Yildirim-Buharalioglu G Bond M Sala-Newby GB Hindmarch CC Newby AC. Regulation of Epigenetic Modifiers, Including KDM6B, by Interferon-gamma and Interleukin-4 in Human Macrophages. Front Immunol (2017) 8:92. 10.3389/fimmu.2017.00092 28228757 PMC5296311

[29] Kruidenier L Chung CW Cheng Z Liddle J Che K Joberty G. A selective jumonji H3K27 demethylase inhibitor modulates the proinflammatory macrophage response. Nature (2012) 488(7411):404–8. 10.1038/nature11262 PMC4691848 22842901

[30] Tausendschon M Dehne N Brune B. Hypoxia causes epigenetic gene regulation in macrophages by attenuating Jumonji histone demethylase activity. Cytokine (2011) 53(2):256–62. 10.1016/j.cyto.2010.11.002 21131212

[31] Osawa T Tsuchida R Muramatsu M Shimamura T Wang F Suehiro J. Inhibition of histone demethylase JMJD1A improves anti-angiogenic therapy and reduces tumor-associated macrophages. Cancer Res (2013) 73(10):3019–28. 10.1158/0008-5472.CAN-12-3231 23492365

[32] Movahedi K Schoonooghe S Laoui D Houbracken I Waelput W Breckpot K. Nanobody-based targeting of the macrophage mannose receptor for effective in vivo imaging of tumor-associated macrophages. Cancer Res (2012) 72(16):4165–77. 10.1158/0008-5472.CAN-11-2994 22719068

[33] Feng D Sangster-Guity N Stone R Korczeniewska J Mancl ME Fitzgerald-Bocarsly P. Differential requirement of histone acetylase and deacetylase activities for IRF5-mediated proinflammatory cytokine expression. J Immunol (2010) 185(10):6003–12. 10.4049/jimmunol.1000482 PMC3233222 20935208

[34] Gnanaprakasam JNR Estrada-Muniz E Vega L. The anacardic 6-pentadecyl salicylic acid induces macrophage activation via the phosphorylation of ERK1/2, JNK, P38 kinases and NF-kappaB. Int Immunopharmacol (2015) 29(2):808–17. 10.1016/j.intimp.2015.08.038 26371858

[35] Wu C Li A Hu J Kang J. Histone deacetylase 2 is essential for LPS-induced inflammatory responses in macrophages. Immunol Cell Biol (2019) 97(1):72–84. 10.1111/imcb.12203 30207412 PMC7379312

[36] Chen X Barozzi I Termanini A Prosperini E Recchiuti A Dalli J. Requirement for the histone deacetylase Hdac3 for the inflammatory gene expression program in macrophages. Proc Natl Acad Sci USA (2012) 109(42):E2865–74. 10.1073/pnas.1121131109 PMC3479529 22802645

[37] Mullican SE Gaddis CA Alenghat T Nair MG Giacomin PR Everett LJ. Histone deacetylase 3 is an epigenomic brake in macrophage alternative activation. Genes Dev (2011) 25(23):2480–8. 10.1101/gad.175950.111 PMC3243058 22156208

[38] Wang B Liu TY Lai CH Rao YH Choi MC Chi JT. Glycolysis-dependent histone deacetylase 4 degradation regulates inflammatory cytokine production. Mol Biol Cell (2014) 25(21):3300–7. 10.1091/mbc.E13-12-0757 PMC4214777 25187650

[39] Edderkaoui M Xu S Chheda C Morvaridi S Hu RW Grippo PJ. HDAC3 mediates smoking-induced pancreatic cancer. Oncotarget (2016) 7(7):7747–60. 10.18632/oncotarget.6820 PMC4884951 26745602

[40] Tran K Risingsong R Royce DB Williams CR Sporn MB Pioli PA. The combination of the histone deacetylase inhibitor vorinostat and synthetic triterpenoids reduces tumorigenesis in mouse models of cancer. Carcinogenesis (2013) 34(1):199–210. 10.1093/carcin/bgs319 23042302 PMC3534195

[41] Halili MA Andrews MR Labzin LI Schroder K Matthias G Cao C. Differential effects of selective HDAC inhibitors on macrophage inflammatory responses to the Toll-like receptor 4 agonist LPS. J Leukoc Biol (2010) 87(6):1103–14. 10.1189/jlb.0509363 20200406

[42] Yang X Wang X Liu D Yu L Xue B Shi H. Epigenetic regulation of macrophage polarization by DNA methyltransferase 3b. Mol Endocrinol (2014) 28(4):565–74. 10.1210/me.2013-1293 PMC3968399 24597547

[43] Cheng C Huang C Ma TT Bian EB He Y Zhang L. SOCS1 hypermethylation mediated by DNMT1 is associated with lipopolysaccharide-induced inflammatory cytokines in macrophages. Toxicol Lett (2014) 225(3):488–97. 10.1016/j.toxlet.2013.12.023 24440346

[44] Travers M Brown SM Dunworth M Holbert CE Wiehagen KR Bachman KE. DFMO and 5-Azacytidine Increase M1 Macrophages in the Tumor Microenvironment of Murine Ovarian Cancer. Cancer Res (2019) 79(13):3445–54. 10.1158/0008-5472.CAN-18-4018 PMC6606334 31088836

[45] Laoui D Keirsse J Morias Y Van Overmeire E Geeraerts X Elkrim Y. The tumour microenvironment harbours ontogenically distinct dendritic cell populations with opposing effects on tumour immunity. Nat Commun (2016) 7:13720. 10.1038/ncomms13720 28008905 PMC5196231

[46] Schlitzer A Zhang W Song M Ma X. Recent advances in understanding dendritic cell development, classification, and phenotype. F1000Res (2018) 7:1558. 10.12688/f1000research.14793.1 PMC6173131 30345015

[47] Goyvaerts C De Groeve K Dingemans J Van Lint S Robays L Heirman C. Development of the Nanobody display technology to target lentiviral vectors to antigen-presenting cells. Gene Ther (2012) 19(12):1133–40. 10.1038/gt.2011.206 PMC3520013 22241177

[48] Lin A Schildknecht A Nguyen LT Ohashi PS. Dendritic cells integrate signals from the tumor microenvironment to modulate immunity and tumor growth. Immunol Lett (2010) 127(2):77–84. 10.1016/j.imlet.2009.09.003 19778555

[49] Schlitzer A Sivakamasundari V Chen J Sumatoh HR Schreuder J Lum J. Identification of cDC1- and cDC2-committed DC progenitors reveals early lineage priming at the common DC progenitor stage in the bone marrow. Nat Immunol (2015) 16(7):718–28. 10.1038/ni.3200 26054720

[50] Vu Manh TP Bertho N Hosmalin A Schwartz-Cornil I Dalod M. Investigating Evolutionary Conservation of Dendritic Cell Subset Identity and Functions. Front Immunol (2015) 6:260. 10.3389/fimmu.2015.00260 26082777 PMC4451681

[51] Cancel JC Crozat K Dalod M Mattiuz R. Are Conventional Type 1 Dendritic Cells Critical for Protective Antitumor Immunity and How? Front Immunol (2019) 10:9. 10.3389/fimmu.2019.00009 30809220 PMC6379659

[52] Bottcher JP Bonavita E Chakravarty P Blees H Cabeza-Cabrerizo M Sammicheli S. NK Cells Stimulate Recruitment of cDC1 into the Tumor Microenvironment Promoting Cancer Immune Control. Cell (2018) 172(5):1022–37.e14. 10.1016/j.cell.2018.01.004 29429633 PMC5847168

[53] Barry KC Hsu J Broz ML Cueto FJ Binnewies M Combes AJ. A natural killer-dendritic cell axis defines checkpoint therapy-responsive tumor microenvironments. Nat Med (2018) 24(8):1178–91. 10.1038/s41591-018-0085-8 PMC6475503 29942093

[54] Roberts EW Broz ML Binnewies M Headley MB Nelson AE Wolf DM. Critical Role for CD103(+)/CD141(+) Dendritic Cells Bearing CCR7 for Tumor Antigen Trafficking and Priming of T Cell Immunity in Melanoma. Cancer Cell (2016) 30(2):324–36. 10.1016/j.ccell.2016.06.003 PMC5374862 27424807

[55] Michea P Noel F Zakine E Czerwinska U Sirven P Abouzid O. Adjustment of dendritic cells to the breast-cancer microenvironment is subset specific. Nat Immunol (2018) 19(8):885–97. 10.1038/s41590-018-0145-8 30013147

[56] Guilliams M Dutertre CA Scott CL McGovern N Sichien D Chakarov S. Unsupervised High-Dimensional Analysis Aligns Dendritic Cells across Tissues and Species. Immunity (2016) 45(3):669–84. 10.1016/j.immuni.2016.08.015 PMC5040826 27637149

[57] Najafi S Mirshafiey A. The role of T helper 17 and regulatory T cells in tumor microenvironment. Immunopharmacol Immunotoxicol (2019) 41(1):16–24. 10.1080/08923973.2019.1566925 30714422

[58] Norian LA Rodriguez PC O’Mara LA Zabaleta J Ochoa AC Cella M. Tumor-infiltrating regulatory dendritic cells inhibit CD8+ T cell function via L-arginine metabolism. Cancer Res (2009) 69(7):3086–94. 10.1158/0008-5472.CAN-08-2826 PMC2848068 19293186

[59] Hochrein H O’Keeffe M Wagner H. Human and mouse plasmacytoid dendritic cells. Hum Immunol (2002) 63(12):1103–10. 10.1016/s0198-8859(02)00748-6 12480253

[60] Conrad C Gregorio J Wang YH Ito T Meller S Hanabuchi S. Plasmacytoid dendritic cells promote immunosuppression in ovarian cancer via ICOS costimulation of Foxp3(+) T-regulatory cells. Cancer Res (2012) 72(20):5240–9. 10.1158/0008-5472.CAN-12-2271 PMC3652570 22850422

[61] Treilleux I Blay JY Bendriss-Vermare N Ray-Coquard I Bachelot T Guastalla JP. Dendritic cell infiltration and prognosis of early stage breast cancer. Clin Cancer Res (2004) 10(22):7466–74. 10.1158/1078-0432.CCR-04-0684 15569976

[62] Lavin Y Kobayashi S Leader A Amir ED Elefant N Bigenwald C. Innate Immune Landscape in Early Lung Adenocarcinoma by Paired Single-Cell Analyses. Cell (2017) 169(4):750–65 e17. 10.1016/j.cell.2017.04.014 28475900 PMC5737939

[63] Wu J Li S Yang Y Zhu S Zhang M Qiao Y. TLR-activated plasmacytoid dendritic cells inhibit breast cancer cell growth in vitro and in vivo. Oncotarget (2017) 8(7):11708–18. 10.18632/oncotarget.14315 PMC5355297 28052019

[64] Guilliams M Ginhoux F Jakubzick C Naik SH Onai N Schraml BU. Dendritic cells, monocytes and macrophages: a unified nomenclature based on ontogeny. Nat Rev Immunol (2014) 14(8):571–8. 10.1038/nri3712 PMC4638219 25033907

[65] Leon B Ardavin C. Monocyte-derived dendritic cells in innate and adaptive immunity. Immunol Cell Biol (2008) 86(4):320–4. 10.1038/icb.2008.14 18362945

[66] Huber A Dammeijer F Aerts J Vroman H. Current State of Dendritic Cell-Based Immunotherapy: Opportunities for in vitro Antigen Loading of Different DC Subsets? Front Immunol (2018) 9:2804. 10.3389/fimmu.2018.02804 30559743 PMC6287551

[67] Boukhaled GM Corrado M Guak H Krawczyk CM. Chromatin Architecture as an Essential Determinant of Dendritic Cell Function. Front Immunol (2019) 10:1119. 10.3389/fimmu.2019.01119 31214161 PMC6557980

[68] Tian Y Meng L Zhang Y. Epigenetic Regulation of Dendritic Cell Development and Function. Cancer J (2017) 23(5):302–7. 10.1097/PPO.0000000000000280 PMC6252173 28926431

[69] Pacis A Tailleux L Morin AM Lambourne J MacIsaac JL Yotova V. Bacterial infection remodels the DNA methylation landscape of human dendritic cells. Genome Res (2015) 25(12):1801–11. 10.1101/gr.192005.115 PMC4665002 26392366

[70] Fang TC Schaefer U Mecklenbrauker I Stienen A Dewell S Chen MS. Histone H3 lysine 9 di-methylation as an epigenetic signature of the interferon response. J Exp Med (2012) 209(4):661–9. 10.1084/jem.20112343 PMC3328357 22412156

[71] Kuo CH Yang SN Tsai YG Hsieh CC Liao WT Chen LC. Long-acting beta2-adrenoreceptor agonists suppress type 1 interferon expression in human plasmacytoid dendritic cells via epigenetic regulation. Pulm Pharmacol Ther (2018) 48:37–45. 10.1016/j.pupt.2017.10.004 28987803

[72] Donas C Carrasco M Fritz M Prado C Tejon G Osorio-Barrios F. The histone demethylase inhibitor GSK-J4 limits inflammation through the induction of a tolerogenic phenotype on DCs. J Autoimmun (2016) 75:105–17. 10.1016/j.jaut.2016.07.011 27528513

[73] Jin J Xie X Xiao Y Hu H Zou Q Cheng X. Epigenetic regulation of the expression of Il12 and Il23 and autoimmune inflammation by the deubiquitinase Trabid. Nat Immunol (2016) 17(3):259–68. 10.1038/ni.3347 PMC4755875 26808229

[74] Huang Y Min S Lui Y Sun J Su X Liu Y. Global mapping of H3K4me3 and H3K27me3 reveals chromatin state-based regulation of human monocyte-derived dendritic cells in different environments. Genes Immun (2012) 13(4):311–20. 10.1038/gene.2011.87 22278394

[75] Zhou Z Chen H Xie R Wang H Li S Xu Q. Epigenetically modulated FOXM1 suppresses dendritic cell maturation in pancreatic cancer and colon cancer. Mol Oncol (2019) 13(4):873–93. 10.1002/1878-0261.12443 PMC6441919 30628173

[76] Choi YE Yu HN Yoon CH Bae YS. Tumor-mediated down-regulation of MHC class II in DC development is attributable to the epigenetic control of the CIITA type I promoter. Eur J Immunol (2009) 39(3):858–68. 10.1002/eji.200838674 19224634

[77] Yang Q Wei J Zhong L Shi M Zhou P Zuo S. Cross talk between histone deacetylase 4 and STAT6 in the transcriptional regulation of arginase 1 during mouse dendritic cell differentiation. Mol Cell Biol (2015) 35(1):63–75. 10.1128/MCB.00805-14 25332236 PMC4295380

[78] Chauvistre H Kustermann C Rehage N Klisch T Mitzka S Felker P. Dendritic cell development requires histone deacetylase activity. Eur J Immunol (2014) 44(8):2478–88. 10.1002/eji.201344150 PMC4209797 24810486

[79] Shen L Orillion A Pili R. Histone deacetylase inhibitors as immunomodulators in cancer therapeutics. Epigenomics (2016) 8(3):415–28. 10.2217/epi.15.118 26950532

[80] Song W Tai YT Tian Z Hideshima T Chauhan D Nanjappa P. HDAC inhibition by LBH589 affects the phenotype and function of human myeloid dendritic cells. Leukemia (2011) 25(1):161–8. 10.1038/leu.2010.244 PMC3839585 21102427

[81] Vento-Tormo R Company C Rodriguez-Ubreva J de la Rica L Urquiza JM Javierre BM. IL-4 orchestrates STAT6-mediated DNA demethylation leading to dendritic cell differentiation. Genome Biol (2016) 17:4. 10.1186/s13059-015-0863-2 26758199 PMC4711003

[82] Zhang X Ulm A Somineni HK Oh S Weirauch MT Zhang HX. DNA methylation dynamics during ex vivo differentiation and maturation of human dendritic cells. Epigenet Chromatin (2014) 7:21. 10.1186/1756-8935-7-21 PMC4144987 25161698

[83] Ma S Wan X Deng Z Shi L Hao C Zhou Z. Epigenetic regulator CXXC5 recruits DNA demethylase Tet2 to regulate TLR7/9-elicited IFN response in pDCs. J Exp Med (2017) 214(5):1471–91. 10.1084/jem.20161149 PMC5413332 28416650

[84] Frikeche J Clavert A Delaunay J Brissot E Gregoire M Gaugler B. Impact of the hypomethylating agent 5-azacytidine on dendritic cells function. Exp Hematol (2011) 39(11):1056–63. 10.1016/j.exphem.2011.08.004 21856273

[85] Fragale A Romagnoli G Licursi V Buoncervello M Del Vecchio G Giuliani C. Antitumor Effects of Epidrug/IFNalpha Combination Driven by Modulated Gene Signatures in Both Colorectal Cancer and Dendritic Cells. Cancer Immunol Res (2017) 5(7):604–16. 10.1158/2326-6066.CIR-17-0080 28615266

[86] De Vlaeminck Y Gonzalez-Rascon A Goyvaerts C Breckpot K. Cancer-Associated Myeloid Regulatory Cells. Front Immunol (2016) 7:113. 10.3389/fimmu.2016.00113 27065074 PMC4810015

[87] Ostrand-Rosenberg S Sinha P Beury DW Clements VK. Cross-talk between myeloid-derived suppressor cells (MDSC), macrophages, and dendritic cells enhances tumor-induced immune suppression. Semin Cancer Biol (2012) 22(4):275–81. 10.1016/j.semcancer.2012.01.011 PMC3701942 22313874

[88] Pan PY Ma G Weber KJ Ozao-Choy J Wang G Yin B. Immune stimulatory receptor CD40 is required for T-cell suppression and T regulatory cell activation mediated by myeloid-derived suppressor cells in cancer. Cancer Res (2010) 70(1):99–108. 10.1158/0008-5472.CAN-09-1882 19996287 PMC2805053

[89] Huang S Wang Z Zhou J Huang J Zhou L Luo J. EZH2 Inhibitor GSK126 Suppresses Antitumor Immunity by Driving Production of Myeloid-Derived Suppressor Cells. Cancer Res (2019) 79(8):2009–20. 10.1158/0008-5472.CAN-18-2395 30737232

[90] Zhou J Huang S Wang Z Huang J Xu L Tang X. Targeting EZH2 histone methyltransferase activity alleviates experimental intestinal inflammation. Nat Commun (2019) 10(1):2427. 10.1038/s41467-019-10176-2 31160593 PMC6547712

[91] Youn JI Kumar V Collazo M Nefedova Y Condamine T Cheng P. Epigenetic silencing of retinoblastoma gene regulates pathologic differentiation of myeloid cells in cancer. Nat Immunol (2013) 14(3):211–20. 10.1038/ni.2526 PMC3578019 23354483

[92] Sahakian E Powers JJ Chen J Deng SL Cheng F Distler A. Histone deacetylase 11: A novel epigenetic regulator of myeloid derived suppressor cell expansion and function. Mol Immunol (2015) 63(2):579–85. 10.1016/j.molimm.2014.08.002 PMC4252813 25155994

[93] de Almeida Nagata DE Chiang EY Jhunjhunwala S Caplazi P Arumugam V Modrusan Z. Regulation of Tumor-Associated Myeloid Cell Activity by CBP/EP300 Bromodomain Modulation of H3K27 Acetylation. Cell Rep (2019) 27(1):269–81.e4. 10.1016/j.celrep.2019.03.008 30943407

[94] Xie Z Ago Y Okada N Tachibana M. Valproic acid attenuates immunosuppressive function of myeloid-derived suppressor cells. J Pharmacol Sci (2018) 137(4):359–65. 10.1016/j.jphs.2018.06.014 30177294

[95] Wang HF Ning F Liu ZC Wu L Li ZQ Qi YF. Histone deacetylase inhibitors deplete myeloid-derived suppressor cells induced by 4T1 mammary tumors in vivo and in vitro. Cancer Immunol Immunother (2017) 66(3):355–66. 10.1007/s00262-016-1935-1 PMC11028551 27915371

[96] Orillion A Hashimoto A Damayanti N Shen L Adelaiye-Ogala R Arisa S. Entinostat Neutralizes Myeloid-Derived Suppressor Cells and Enhances the Antitumor Effect of PD-1 Inhibition in Murine Models of Lung and Renal Cell Carcinoma. Clin Cancer Res (2017) 23(17):5187–201. 10.1158/1078-0432.CCR-17-0741 PMC5723438 28698201

[97] Christmas BJ Rafie CI Hopkins AC Scott BA Ma HS Cruz KA. Entinostat Converts Immune-Resistant Breast and Pancreatic Cancers into Checkpoint-Responsive Tumors by Reprogramming Tumor-Infiltrating MDSCs. Cancer Immunol Res (2018) 6(12):1561–77. 10.1158/2326-6066.CIR-18-0070 PMC6279584 30341213

[98] Rosborough BR Castellaneta A Natarajan S Thomson AW Turnquist HR. Histone deacetylase inhibition facilitates GM-CSF-mediated expansion of myeloid-derived suppressor cells in vitro and in vivo. J Leukoc Biol (2012) 91(5):701–9. 10.1189/jlb.0311119 PMC4046249 22028329

[99] Rodriguez-Ubreva J Catala-Moll F Obermajer N Alvarez-Errico D Ramirez RN Company C. Prostaglandin E2 Leads to the Acquisition of DNMT3A-Dependent Tolerogenic Functions in Human Myeloid-Derived Suppressor Cells. Cell Rep (2017) 21(1):154–67. 10.1016/j.celrep.2017.09.018 28978469

[100] Zhou J Shen Q Lin H Hu L Li G Zhang X. Decitabine shows potent anti-myeloma activity by depleting monocytic myeloid-derived suppressor cells in the myeloma microenvironment. J Cancer Res Clin Oncol (2019) 145(2):329–36. 10.1007/s00432-018-2790-6 PMC11810327 30426212

[101] Zhou J Yao Y Shen Q Li G Hu L Zhang X. Demethylating agent decitabine disrupts tumor-induced immune tolerance by depleting myeloid-derived suppressor cells. J Cancer Res Clin Oncol (2017) 143(8):1371–80. 10.1007/s00432-017-2394-6 PMC11819064 28321548

[102] Daurkin I Eruslanov E Vieweg J Kusmartsev S. Generation of antigen-presenting cells from tumor-infiltrated CD11b myeloid cells with DNA demethylating agent 5-aza-2’-deoxycytidine. Cancer Immunol Immunother (2010) 59(5):697–706. 10.1007/s00262-009-0786-4 19882154 PMC11030737

[103] Schmidl C Delacher M Huehn J Feuerer M. Epigenetic mechanisms regulating T-cell responses. J Allergy Clin Immunol (2018) 142(3):728–43. 10.1016/j.jaci.2018.07.014 30195378

[104] Coulie PG Van den Eynde BJ van der Bruggen P Boon T. Tumour antigens recognized by T lymphocytes: at the core of cancer immunotherapy. Nat Rev Cancer (2014) 14(2):135–46. 10.1038/nrc3670 24457417

[105] Durek P Nordstrom K Gasparoni G Salhab A Kressler C de Almeida M. Epigenomic Profiling of Human CD4(+) T Cells Supports a Linear Differentiation Model and Highlights Molecular Regulators of Memory Development. Immunity (2016) 45(5):1148–61. 10.1016/j.immuni.2016.10.022 27851915

[106] LaMere SA Thompson RC Komori HK Mark A Salomon DR. Promoter H3K4 methylation dynamically reinforces activation-induced pathways in human CD4 T cells. Genes Immun (2016) 17(5):283–97. 10.1038/gene.2016.19 PMC4956548 27170561

[107] LaMere SA Thompson RC Meng X Komori HK Mark A Salomon DR. H3K27 Methylation Dynamics during CD4 T Cell Activation: Regulation of JAK/STAT and IL12RB2 Expression by JMJD3. J Immunol (2017) 199(9):3158–75. 10.4049/jimmunol.1700475 PMC5679303 28947543

[108] Barski A Cuddapah S Kartashov AV Liu C Imamichi H Yang W. Rapid Recall Ability of Memory T cells is Encoded in their Epigenome. Sci Rep (2017) 7:39785. 10.1038/srep39785 28054639 PMC5215294

[109] Vahedi G Kanno Y Furumoto Y Jiang K Parker SC Erdos MR. Super-enhancers delineate disease-associated regulatory nodes in T cells. Nature (2015) 520(7548):558–62. 10.1038/nature14154 PMC4409450 25686607

[110] Ciofani M Madar A Galan C Sellars M Mace K Pauli F. A validated regulatory network for Th17 cell specification. Cell (2012) 151(2):289–303. 10.1016/j.cell.2012.09.016 23021777 PMC3503487

[111] Hawkins RD Larjo A Tripathi SK Wagner U Luu Y Lonnberg T. Global chromatin state analysis reveals lineage-specific enhancers during the initiation of human T helper 1 and T helper 2 cell polarization. Immunity (2013) 38(6):1271–84. 10.1016/j.immuni.2013.05.011 PMC4607036 23791644

[112] Adoue V Binet B Malbec A Fourquet J Romagnoli P van Meerwijk JPM. The Histone Methyltransferase SETDB1 Controls T Helper Cell Lineage Integrity by Repressing Endogenous Retroviruses. Immunity (2019) 50(3):629–44.e8. 10.1016/j.immuni.2019.01.003 30737147

[113] Tumes DJ Onodera A Suzuki A Shinoda K Endo Y Iwamura C. The polycomb protein Ezh2 regulates differentiation and plasticity of CD4(+) T helper type 1 and type 2 cells. Immunity (2013) 39(5):819–32. 10.1016/j.immuni.2013.09.012 24238339

[114] Li Q Zou J Wang M Ding X Chepelev I Zhou X. Critical role of histone demethylase Jmjd3 in the regulation of CD4+ T-cell differentiation. Nat Commun (2014) 5:5780. 10.1038/ncomms6780 25531312 PMC4274750

[115] Grausenburger R Bilic I Boucheron N Zupkovitz G El-Housseiny L Tschismarov R. Conditional deletion of histone deacetylase 1 in T cells leads to enhanced airway inflammation and increased Th2 cytokine production. J Immunol (2010) 185(6):3489–97. 10.4049/jimmunol.0903610 PMC3175530 20702731

[116] Woods DM Woan KV Cheng F Sodre AL Wang D Wu Y. T cells lacking HDAC11 have increased effector functions and mediate enhanced alloreactivity in a murine model. Blood (2017) 130(2):146–55. 10.1182/blood-2016-08-731505 PMC5510785 28550044

[117] Tomasoni R Basso V Pilipow K Sitia G Saccani S Agresti A. Rapamycin-sensitive signals control TCR/CD28-driven Ifng, Il4 and Foxp3 transcription and promoter region methylation. Eur J Immunol (2011) 41(7):2086–96. 10.1002/eji.201041130 21480212

[118] Thomas RM Gamper CJ Ladle BH Powell JD Wells AD. De novo DNA methylation is required to restrict T helper lineage plasticity. J Biol Chem (2012) 287(27):22900–9. 10.1074/jbc.M111.312785 PMC3391093 22584578

[119] Winders BR Schwartz RH Bruniquel D. A distinct region of the murine IFN-gamma promoter is hypomethylated from early T cell development through mature naive and Th1 cell differentiation, but is hypermethylated in Th2 cells. J Immunol (2004) 173(12):7377–84. 10.4049/jimmunol.173.12.7377 15585862

[120] Zheng Y Josefowicz SZ Kas A Chu TT Gavin MA Rudensky AY. Genome-wide analysis of Foxp3 target genes in developing and mature regulatory T cells. Nature (2007) 445(7130):936–40. 10.1038/nature05563 17237761

[121] Hnisz D Abraham BJ Lee TI Lau A Saint-Andre V Sigova AA. Super-enhancers in the control of cell identity and disease. Cell (2013) 155(4):934–47. 10.1016/j.cell.2013.09.053 PMC3841062 24119843

[122] Kitagawa Y Ohkura N Kidani Y Vandenbon A Hirota K Kawakami R. Guidance of regulatory T cell development by Satb1-dependent super-enhancer establishment. Nat Immunol (2017) 18(2):173–83. 10.1038/ni.3646 PMC5582804 27992401

[123] Ohkura N Yasumizu Y Kitagawa Y Tanaka A Nakamura Y Motooka D. Regulatory T Cell-Specific Epigenomic Region Variants Are a Key Determinant of Susceptibility to Common Autoimmune Diseases. Immunity (2020) 52(6):1119–32.e4. 10.1016/j.immuni.2020.04.006 32362325

[124] Antignano F Burrows K Hughes MR Han JM Kron KJ Penrod NM. Methyltransferase G9A regulates T cell differentiation during murine intestinal inflammation. J Clin Invest (2014) 124(5):1945–55. 10.1172/JCI69592 PMC4001530 24667637

[125] Floess S Freyer J Siewert C Baron U Olek S Polansky J. Epigenetic control of the foxp3 locus in regulatory T cells. PloS Biol (2007) 5(2):e38. 10.1371/journal.pbio.0050038 17298177 PMC1783672

[126] Xiao Y Nagai Y Deng G Ohtani T Zhu Z Zhou Z. Dynamic interactions between TIP60 and p300 regulate FOXP3 function through a structural switch defined by a single lysine on TIP60. Cell Rep (2014) 7(5):1471–80. 10.1016/j.celrep.2014.04.021 PMC4064594 24835996

[127] Kwon HS Lim HW Wu J Schnolzer M Verdin E Ott M. Three novel acetylation sites in the Foxp3 transcription factor regulate the suppressive activity of regulatory T cells. J Immunol (2012) 188(6):2712–21. 10.4049/jimmunol.1100903 PMC3478122 22312127

[128] von Knethen A Heinicke U Weigert A Zacharowski K Brune B. Histone Deacetylation Inhibitors as Modulators of Regulatory T Cells. Int J Mol Sci (2020) 21(7):2356. 10.3390/ijms21072356 PMC7177531 32235291

[129] Xiong Y Khanna S Grzenda AL Sarmento OF Svingen PA Lomberk GA. Polycomb antagonizes p300/CREB-binding protein-associated factor to silence FOXP3 in a Kruppel-like factor-dependent manner. J Biol Chem (2012) 287(41):34372–85. 10.1074/jbc.M111.325332 PMC3464543 22896699

[130] Zheng Y Josefowicz S Chaudhry A Peng XP Forbush K Rudensky AY. Role of conserved non-coding DNA elements in the Foxp3 gene in regulatory T-cell fate. Nature (2010) 463(7282):808–12. 10.1038/nature08750 PMC2884187 20072126

[131] Feng Y Arvey A Chinen T van der Veeken J Gasteiger G Rudensky AY. Control of the inheritance of regulatory T cell identity by a cis element in the Foxp3 locus. Cell (2014) 158(4):749–63. 10.1016/j.cell.2014.07.031 PMC4151558 25126783

[132] Li X Liang Y LeBlanc M Benner C Zheng Y. Function of a Foxp3 cis-element in protecting regulatory T cell identity. Cell (2014) 158(4):734–48. 10.1016/j.cell.2014.07.030 PMC4151505 25126782

[133] Morikawa H Ohkura N Vandenbon A Itoh M Nagao-Sato S Kawaji H. Differential roles of epigenetic changes and Foxp3 expression in regulatory T cell-specific transcriptional regulation. Proc Natl Acad Sci USA (2014) 111(14):5289–94. 10.1073/pnas.1312717110 PMC3986152 24706905

[134] Ohkura N Hamaguchi M Morikawa H Sugimura K Tanaka A Ito Y. T cell receptor stimulation-induced epigenetic changes and Foxp3 expression are independent and complementary events required for Treg cell development. Immunity (2012) 37(5):785–99. 10.1016/j.immuni.2012.09.010 23123060

[135] Wang L Liu Y Beier UH Han R Bhatti TR Akimova T. Foxp3+ T-regulatory cells require DNA methyltransferase 1 expression to prevent development of lethal autoimmunity. Blood (2013) 121(18):3631–9. 10.1182/blood-2012-08-451765 PMC3643763 23444399

[136] Bontkes HJ Ruben JM Alhan C Westers TM Ossenkoppele GJ van de Loosdrecht AA. Azacitidine differentially affects CD4(pos) T-cell polarization in vitro and in vivo in high risk myelodysplastic syndromes. Leuk Res (2012) 36(7):921–30. 10.1016/j.leukres.2012.03.026 22503132

[137] Lu CH Wu CJ Chan CC Nguyen DT Lin KR Lin SJ. DNA Methyltransferase Inhibitor Promotes Human CD4(+)CD25(h)FOXP3(+) Regulatory T Lymphocyte Induction under Suboptimal TCR Stimulation. Front Immunol (2016) 7:488:488. 10.3389/fimmu.2016.00488 27877174 PMC5099256

[138] Stubig T Badbaran A Luetkens T Hildebrandt Y Atanackovic D Binder TM. 5-azacytidine promotes an inhibitory T-cell phenotype and impairs immune mediated antileukemic activity. Mediators Inflammation (2014) 2014:418292. 10.1155/2014/418292 PMC3976863 24757283

[139] Costantini B Kordasti SY Kulasekararaj AG Jiang J Seidl T Abellan PP. The effects of 5-azacytidine on the function and number of regulatory T cells and T-effectors in myelodysplastic syndrome. Haematologica (2013) 98(8):1196–205. 10.3324/haematol.2012.074823 PMC3729899 23242597

[140] Kehrmann J Tatura R Zeschnigk M Probst-Kepper M Geffers R Steinmann J. Impact of 5-aza-2’-deoxycytidine and epigallocatechin-3-gallate for induction of human regulatory T cells. Immunology (2014) 142(3):384–95. 10.1111/imm.12261 PMC4080954 24476360

[141] Landman S Cruijsen M Urbano PCM Huls G van Erp PEJ van Rijssen E. DNA Methyltransferase Inhibition Promotes Th1 Polarization in Human CD4(+)CD25(high) FOXP3(+) Regulatory T Cells but Does Not Affect Their Suppressive Capacity. J Immunol Res (2018) 2018:4973964. 10.1155/2018/4973964 29850630 PMC5924998

[142] Henning AN Roychoudhuri R Restifo NP. Epigenetic control of CD8(+) T cell differentiation. Nat Rev Immunol (2018) 18(5):340–56. 10.1038/nri.2017.146 PMC6327307 29379213

[143] Abdelsamed HA Moustaki A Fan Y Dogra P Ghoneim HE Zebley CC. Human memory CD8 T cell effector potential is epigenetically preserved during in vivo homeostasis. J Exp Med (2017) 214(6):1593–606. 10.1084/jem.20161760 PMC5461005 28490440

[144] Crompton JG Narayanan M Cuddapah S Roychoudhuri R Ji Y Yang W. Lineage relationship of CD8(+) T cell subsets is revealed by progressive changes in the epigenetic landscape. Cell Mol Immunol (2016) 13(4):502–13. 10.1038/cmi.2015.32 PMC4947817 25914936

[145] Youngblood B Hale JS Kissick HT Ahn E Xu X Wieland A. Effector CD8 T cells dedifferentiate into long-lived memory cells. Nature (2017) 552(7685):404–9. 10.1038/nature25144 PMC5965677 29236683

[146] Russ BE Olshanksy M Smallwood HS Li J Denton AE Prier JE. Distinct epigenetic signatures delineate transcriptional programs during virus-specific CD8(+) T cell differentiation. Immunity (2014) 41(5):853–65. 10.1016/j.immuni.2014.11.001 PMC4479393 25517617

[147] Gray SM Amezquita RA Guan T Kleinstein SH Kaech SM. Polycomb Repressive Complex 2-Mediated Chromatin Repression Guides Effector CD8(+) T Cell Terminal Differentiation and Loss of Multipotency. Immunity (2017) 46(4):596–608. 10.1016/j.immuni.2017.03.012 28410989 PMC5457165

[148] Ladle BH Li KP Phillips MJ Pucsek AB Haile A Powell JD. De novo DNA methylation by DNA methyltransferase 3a controls early effector CD8+ T-cell fate decisions following activation. Proc Natl Acad Sci USA (2016) 113(38):10631–6. 10.1073/pnas.1524490113 PMC5035851 27582468

[149] Sen DR Kaminski J Barnitz RA Kurachi M Gerdemann U Yates KB. The epigenetic landscape of T cell exhaustion. Science (2016) 354(6316):1165–9. 10.1126/science.aae0491 PMC5497589 27789799

[150] Mognol GP Spreafico R Wong V Scott-Browne JP Togher S Hoffmann A. Exhaustion-associated regulatory regions in CD8(+) tumor-infiltrating T cells. Proc Natl Acad Sci USA (2017) 114(13):E2776–E85. 10.1073/pnas.1620498114 PMC5380094 28283662

[151] Pauken KE Sammons MA Odorizzi PM Manne S Godec J Khan O. Epigenetic stability of exhausted T cells limits durability of reinvigoration by PD-1 blockade. Science (2016) 354(6316):1160–5. 10.1126/science.aaf2807 PMC5484795 27789795

[152] Philip M Fairchild L Sun L Horste EL Camara S Shakiba M. Chromatin states define tumour-specific T cell dysfunction and reprogramming. Nature (2017) 545(7655):452–6. 10.1038/nature22367 PMC5693219 28514453

[153] Khan O Giles JR McDonald S Manne S Ngiow SF Patel KP. TOX transcriptionally and epigenetically programs CD8(+) T cell exhaustion. Nature (2019) 571(7764):211–8. 10.1038/s41586-019-1325-x PMC6713202 31207603

[154] Ghoneim HE Fan Y Moustaki A Abdelsamed HA Dash P Dogra P. De Novo Epigenetic Programs Inhibit PD-1 Blockade-Mediated T Cell Rejuvenation. Cell (2017) 170(1):142–57 e19. 10.1016/j.cell.2017.06.007 28648661 PMC5568784

[155] Abel AM Yang C Thakar MS Malarkannan S. Natural Killer Cells: Development, Maturation, and Clinical Utilization. Front Immunol (2018) 9:1869. 10.3389/fimmu.2018.01869 30150991 PMC6099181

[156] Paul S Lal G. The Molecular Mechanism of Natural Killer Cells Function and Its Importance in Cancer Immunotherapy. Front Immunol (2017) 8:1124. 10.3389/fimmu.2017.01124 28955340 PMC5601256

[157] Bugide S Janostiak R Wajapeyee N. Epigenetic Mechanisms Dictating Eradication of Cancer by Natural Killer Cells. Trends Cancer (2018) 4(8):553–66. 10.1016/j.trecan.2018.06.004 PMC6085095 30064663

[158] Yin J Leavenworth JW Li Y Luo Q Xie H Liu X. Ezh2 regulates differentiation and function of natural killer cells through histone methyltransferase activity. Proc Natl Acad Sci USA (2015) 112(52):15988–93. 10.1073/pnas.1521740112 PMC4702963 26668377

[159] Li Y Wang J Yin J Liu X Yu M Li T. Chromatin state dynamics during NK cell activation. Oncotarget (2017) 8(26):41854–65. 10.18632/oncotarget.16688 PMC5522033 28402957

[160] Zhu S Denman CJ Cobanoglu ZS Kiany S Lau CC Gottschalk SM. The narrow-spectrum HDAC inhibitor entinostat enhances NKG2D expression without NK cell toxicity, leading to enhanced recognition of cancer cells. Pharm Res (2015) 32(3):779–92. 10.1007/s11095-013-1231-0 PMC4014531 24203492

[161] Kato N Tanaka J Sugita J Toubai T Miura Y Ibata M. Regulation of the expression of MHC class I-related chain A, B (MICA, MICB) via chromatin remodeling and its impact on the susceptibility of leukemic cells to the cytotoxicity of NKG2D-expressing cells. Leukemia (2007) 21(10):2103–8. 10.1038/sj.leu.2404862 17625602

[162] Conte M De Palma R Altucci L. HDAC inhibitors as epigenetic regulators for cancer immunotherapy. Int J Biochem Cell Biol (2018) 98:65–74. 10.1016/j.biocel.2018.03.004 29535070

[163] Gao XN Lin J Wang LL Yu L. Demethylating treatment suppresses natural killer cell cytolytic activity. Mol Immunol (2009) 46(10):2064–70. 10.1016/j.molimm.2009.02.033 19394699

[164] Dunn GP Old LJ Schreiber RD. The three Es of cancer immunoediting. Annu Rev Immunol (2004) 22:329–60. 10.1146/annurev.immunol.22.012703.104803 15032581

[165] Chen DS Mellman I. Elements of cancer immunity and the cancer-immune set point. Nature (2017) 541(7637):321–30. 10.1038/nature21349 28102259

[166] Bhatia A Kumar Y. Cellular and molecular mechanisms in cancer immune escape: a comprehensive review. Expert Rev Clin Immunol (2014) 10(1):41–62. 10.1586/1744666X.2014.865519 24325346

[167] Galon J Bruni D. Approaches to treat immune hot, altered and cold tumours with combination immunotherapies. Nat Rev Drug Discovery (2019) 18(3):197–218. 10.1038/s41573-018-0007-y 30610226

[168] Nguyen KB Spranger S. Modulation of the immune microenvironment by tumor-intrinsic oncogenic signaling. J Cell Biol (2020) 219(1):e201908224. 10.1083/jcb.201908224 31816057 PMC7039199

[169] Burr ML Sparbier CE Chan KL Chan YC Kersbergen A Lam EYN. An Evolutionarily Conserved Function of Polycomb Silences the MHC Class I Antigen Presentation Pathway and Enables Immune Evasion in Cancer. Cancer Cell (2019) 36(4):385–401.e8. 10.1016/j.ccell.2019.08.008 31564637 PMC6876280

[170] Ennishi D Takata K Beguelin W Duns G Mottok A Farinha P. Molecular and Genetic Characterization of MHC Deficiency Identifies EZH2 as Therapeutic Target for Enhancing Immune Recognition. Cancer Discov (2019) 9(4):546–63. 10.1158/2159-8290.CD-18-1090 30705065

[171] Zingg D Arenas-Ramirez N Sahin D Rosalia RA Antunes AT Haeusel J. The Histone Methyltransferase Ezh2 Controls Mechanisms of Adaptive Resistance to Tumor Immunotherapy. Cell Rep (2017) 20(4):854–67. 10.1016/j.celrep.2017.07.007 28746871

[172] Hamaidia M Gazon H Hoyos C Hoffmann GB Louis R Duysinx B. Inhibition of EZH2 methyltransferase decreases immunoediting of mesothelioma cells by autologous macrophages through a PD-1-dependent mechanism. JCI Insight (2019) 4(18):e128474. 10.1172/jci.insight.128474 PMC6795292 31534051

[173] Jeong SY Lee JH Kim HS Hong SH Cheong CH Kim IK. 3-Deazaadenosine analogues inhibit the production of tumour necrosis factor-alpha in RAW264.7 cells stimulated with lipopolysaccharide. Immunology (1996) 89(4):558–62. 10.1046/j.1365-2567.1996.d01-781.x PMC1456586 9014821

[174] Zhang X Wang Y Yuan J Li N Pei S Xu J. Macrophage/microglial Ezh2 facilitates autoimmune inflammation through inhibition of Socs3. J Exp Med (2018) 215(5):1365–82. 10.1084/jem.20171417 PMC5940261 29626115

[175] Magner WJ Kazim AL Stewart C Romano MA Catalano G Grande C. Activation of MHC class I, II, and CD40 gene expression by histone deacetylase inhibitors. J Immunol (2000) 165(12):7017–24. 10.4049/jimmunol.165.12.7017 11120829

[176] Setiadi AF Omilusik K David MD Seipp RP Hartikainen J Gopaul R. Epigenetic enhancement of antigen processing and presentation promotes immune recognition of tumors. Cancer Res (2008) 68(23):9601–7. 10.1158/0008-5472.CAN-07-5270 19047136

[177] Khan AN Gregorie CJ Tomasi TB. Histone deacetylase inhibitors induce TAP, LMP, Tapasin genes and MHC class I antigen presentation by melanoma cells. Cancer Immunol Immunother (2008) 57(5):647–54. 10.1007/s00262-007-0402-4 PMC3146348 18046553

[178] Dubovsky JA Wang D Powers JJ Berchmans E Smith MA Wright KL. Restoring the functional immunogenicity of chronic lymphocytic leukemia using epigenetic modifiers. Leuk Res (2011) 35(3):394–404. 10.1016/j.leukres.2010.08.001 20863567 PMC4458853

[179] Gameiro SR Malamas AS Tsang KY Ferrone S Hodge JW. Inhibitors of histone deacetylase 1 reverse the immune evasion phenotype to enhance T-cell mediated lysis of prostate and breast carcinoma cells. Oncotarget (2016) 7(7):7390–402. 10.18632/oncotarget.7180 PMC4884926 26862729

[180] Leclercq S Gueugnon F Boutin B Guillot F Blanquart C Rogel A. A 5-aza-2’-deoxycytidine/valproate combination induces cytotoxic T-cell response against mesothelioma. Eur Respir J (2011) 38(5):1105–16. 10.1183/09031936.00081310 21540307

[181] Guerriero JL Sotayo A Ponichtera HE Castrillon JA Pourzia AL Schad S. Class IIa HDAC inhibition reduces breast tumours and metastases through anti-tumour macrophages. Nature (2017) 543(7645):428–+. 10.1038/nature21409 PMC8170529 28273064

[182] Mao W Ghasemzadeh A Freeman ZT Obradovic A Chaimowitz MG Nirschl TR. Immunogenicity of prostate cancer is augmented by BET bromodomain inhibition. J Immunother Cancer (2019) 7(1):277. 10.1186/s40425-019-0758-y 31653272 PMC6814994

[183] Liu K Zhou Z Gao H Yang F Qian Y Jin H. JQ1, a BET-bromodomain inhibitor, inhibits human cancer growth and suppresses PD-L1 expression. Cell Biol Int (2019) 43(6):642–50. 10.1002/cbin.11139 30958600

[184] Zhu H Bengsch F Svoronos N Rutkowski MR Bitler BG Allegrezza MJ. BET Bromodomain Inhibition Promotes Anti-tumor Immunity by Suppressing PD-L1 Expression. Cell Rep (2016) 16(11):2829–37. 10.1016/j.celrep.2016.08.032 PMC5177024 27626654

[185] Erkes DA Field CO Capparelli C Tiago M Purwin TJ Chervoneva I. The next-generation BET inhibitor, PLX51107, delays melanoma growth in a CD8-mediated manner. Pigment Cell Melanoma Res (2019) 32(5):687–96. 10.1111/pcmr.12788 PMC6697571 31063649

[186] Adeegbe DO Liu Y Lizotte PH Kamihara Y Aref AR Almonte C. Synergistic Immunostimulatory Effects and Therapeutic Benefit of Combined Histone Deacetylase and Bromodomain Inhibition in Non-Small Cell Lung Cancer. Cancer Discov (2017) 7(8):852–67. 10.1158/2159-8290.CD-16-1020 PMC5540748 28408401

[187] Galluzzi L Vitale I Warren S Adjemian S Agostinis P Martinez AB. Consensus guidelines for the definition, detection and interpretation of immunogenic cell death. J Immunother Cancer (2020) 8(1):e000337. 10.1136/jitc-2019-000337 32209603 PMC7064135

[188] Khan AN Magner WJ Tomasi TB. An epigenetically altered tumor cell vaccine. Cancer Immunol Immunother (2004) 53(8):748–54. 10.1007/s00262-004-0513-0 PMC11032794 14997346

[189] Khan AN Magner WJ Tomasi TB. An epigenetic vaccine model active in the prevention and treatment of melanoma. J Transl Med (2007) 5:64. 10.1186/1479-5876-5-64 18070359 PMC2231344

[190] Chiappinelli KB Strissel PL Desrichard A Li H Henke C Akman B. Inhibiting DNA Methylation Causes an Interferon Response in Cancer via dsRNA Including Endogenous Retroviruses. Cell (2015) 162(5):974–86. 10.1016/j.cell.2015.07.011 PMC4556003 26317466

[191] Roulois D Loo Yau H Singhania R Wang Y Danesh A Shen SY. DNA-Demethylating Agents Target Colorectal Cancer Cells by Inducing Viral Mimicry by Endogenous Transcripts. Cell (2015) 162(5):961–73. 10.1016/j.cell.2015.07.056 PMC4843502 26317465

[192] Jung H Kim HS Kim JY Sun JM Ahn JS Ahn MJ. DNA methylation loss promotes immune evasion of tumours with high mutation and copy number load. Nat Commun (2019) 10(1):4278. 10.1038/s41467-019-12159-9 31537801 PMC6753140

[193] Liu P Zhao L Loos F Iribarren K Lachkar S Zhou H. Identification of pharmacological agents that induce HMGB1 release. Sci Rep (2017) 7(1):14915. 10.1038/s41598-017-14848-1 29097772 PMC5668281

[194] Sonnemann J Gressmann S Becker S Wittig S Schmudde M Beck JF. The histone deacetylase inhibitor vorinostat induces calreticulin exposure in childhood brain tumour cells in vitro. Cancer Chemother Pharmacol (2010) 66(3):611–6. 10.1007/s00280-010-1302-4 20221600

[195] Wang M Zhao L Tong D Yang L Zhu H Li Q. BET bromodomain inhibitor JQ1 promotes immunogenic cell death in tongue squamous cell carcinoma. Int Immunopharmacol (2019) 76:105921. 10.1016/j.intimp.2019.105921 31600692

[196] Betancur PA Abraham BJ Yiu YY Willingham SB Khameneh F Zarnegar M. A CD47-associated super-enhancer links pro-inflammatory signalling to CD47 upregulation in breast cancer. Nat Commun (2017) 8:14802. 10.1038/ncomms14802 28378740 PMC5382276

[197] Griffiths EA Srivastava P Matsuzaki J Brumberger Z Wang ES Kocent J. NY-ESO-1 Vaccination in Combination with Decitabine Induces Antigen-Specific T-lymphocyte Responses in Patients with Myelodysplastic Syndrome. Clin Cancer Res (2018) 24(5):1019–29. 10.1158/1078-0432.CCR-17-1792 PMC5844797 28947565

[198] Odunsi K Matsuzaki J James SR Mhawech-Fauceglia P Tsuji T Miller A. Epigenetic potentiation of NY-ESO-1 vaccine therapy in human ovarian cancer. Cancer Immunol Res (2014) 2(1):37–49. 10.1158/2326-6066.CIR-13-0126 24535937 PMC3925074

[199] Krishnadas DK Shusterman S Bai F Diller L Sullivan JE Cheerva AC. A phase I trial combining decitabine/dendritic cell vaccine targeting MAGE-A1, MAGE-A3 and NY-ESO-1 for children with relapsed or therapy-refractory neuroblastoma and sarcoma. Cancer Immunol Immunother (2015) 64(10):1251–60. 10.1007/s00262-015-1731-3 PMC11028635 26105625

[200] Lee SY Huang Z Kang TH Soong RS Knoff J Axenfeld E. Histone deacetylase inhibitor AR-42 enhances E7-specific CD8(+) T cell-mediated antitumor immunity induced by therapeutic HPV DNA vaccination. J Mol Med (Berl) (2013) 91(10):1221–31. 10.1007/s00109-013-1054-9 PMC3783646 23715898

[201] Badamchi-Zadeh A Moynihan KD Larocca RA Aid M Provine NM Iampietro MJ. Combined HDAC and BET Inhibition Enhances Melanoma Vaccine Immunogenicity and Efficacy. J Immunol (2018) 201(9):2744–52. 10.4049/jimmunol.1800885 PMC6196294 30249811

[202] Goswami S Apostolou I Zhang J Skepner J Anandhan S Zhang XJ. Modulation of EZH2 expression in T cells improves efficacy of anti-CTLA-4 therapy. J Clin Invest (2018) 128(9):3813–8. 10.1172/Jci99760 PMC6118570 29905573

[203] Qin Y Vasilatos SN Chen L Wu H Cao Z Fu Y. Inhibition of histone lysine-specific demethylase 1 elicits breast tumor immunity and enhances antitumor efficacy of immune checkpoint blockade. Oncogene (2019) 38(3):390–405. 10.1038/s41388-018-0451-5 30111819 PMC6336685

[204] Woods DM Sodre AL Villagra A Sarnaik A Sotomayor EM Weber J. HDAC Inhibition Upregulates PD-1 Ligands in Melanoma and Augments Immunotherapy with PD-1 Blockade. Cancer Immunol Res (2015) 3(12):1375–85. 10.1158/2326-6066.CIR-15-0077-T PMC4674300 26297712

[205] Deng S Hu Q Zhang H Yang F Peng C Huang C. HDAC3 Inhibition Upregulates PD-L1 Expression in B-Cell Lymphomas and Augments the Efficacy of Anti-PD-L1 Therapy. Mol Cancer Ther (2019) 18(5):900–8. 10.1158/1535-7163.MCT-18-1068 30824609

[206] Fukumoto T Fatkhutdinov N Zundell JA Tcyganov EN Nacarelli T Karakashev S. HDAC6 Inhibition Synergizes with Anti-PD-L1 Therapy in ARID1A-Inactivated Ovarian Cancer. Cancer Res (2019) 79(21):5482–9. 10.1158/0008-5472.CAN-19-1302 PMC6825538 31311810

[207] Llopiz D Ruiz M Villanueva L Iglesias T Silva L Egea J. Enhanced anti-tumor efficacy of checkpoint inhibitors in combination with the histone deacetylase inhibitor Belinostat in a murine hepatocellular carcinoma model. Cancer Immunol Immunother (2019) 68(3):379–93. 10.1007/s00262-018-2283-0 PMC11028337 30547218

[208] Briere D Sudhakar N Woods DM Hallin J Engstrom LD Aranda R. The class I/IV HDAC inhibitor mocetinostat increases tumor antigen presentation, decreases immune suppressive cell types and augments checkpoint inhibitor therapy. Cancer Immunol Immunother (2018) 67(3):381–92. 10.1007/s00262-017-2091-y PMC11028326 29124315

[209] Zheng H Zhao W Yan C Watson CC Massengill M Xie M. HDAC Inhibitors Enhance T-Cell Chemokine Expression and Augment Response to PD-1 Immunotherapy in Lung Adenocarcinoma. Clin Cancer Res (2016) 22(16):4119–32. 10.1158/1078-0432.CCR-15-2584 PMC4987196 26964571

[210] Hicks KC Fantini M Donahue RN Schwab A Knudson KM Tritsch SR. Epigenetic priming of both tumor and NK cells augments antibody-dependent cellular cytotoxicity elicited by the anti-PD-L1 antibody avelumab against multiple carcinoma cell types. Oncoimmunology (2018) 7(11):e1466018. 10.1080/2162402X.2018.1466018 30377559 PMC6205056

[211] Lai Q Wang H Li A Xu Y Tang L Chen Q. Decitibine improve the efficiency of anti-PD-1 therapy via activating the response to IFN/PD-L1 signal of lung cancer cells. Oncogene (2018) 37(17):2302–12. 10.1038/s41388-018-0125-3 29422611

[212] Luo N Nixon MJ Gonzalez-Ericsson PI Sanchez V Opalenik SR Li H. DNA methyltransferase inhibition upregulates MHC-I to potentiate cytotoxic T lymphocyte responses in breast cancer. Nat Commun (2018) 9(1):248. 10.1038/s41467-017-02630-w 29339738 PMC5770411

[213] Kim K Skora AD Li Z Liu Q Tam AJ Blosser RL. Eradication of metastatic mouse cancers resistant to immune checkpoint blockade by suppression of myeloid-derived cells. Proc Natl Acad Sci USA (2014) 111(32):11774–9. 10.1073/pnas.1410626111 PMC4136565 25071169

[214] Guo ZS Hong JA Irvine KR Chen GA Spiess PJ Liu Y. De novo induction of a cancer/testis antigen by 5-aza-2’-deoxycytidine augments adoptive immunotherapy in a murine tumor model. Cancer Res (2006) 66(2):1105–13. 10.1158/0008-5472.CAN-05-3020 PMC2242843 16424047

[215] Toor AA Payne KK Chung HM Sabo RT Hazlett AF Kmieciak M. Epigenetic induction of adaptive immune response in multiple myeloma: sequential azacitidine and lenalidomide generate cancer testis antigen-specific cellular immunity. Br J Haematol (2012) 158(6):700–11. 10.1111/j.1365-2141.2012.09225.x PMC4968567 22816680

[216] Terracina KP Graham LJ Payne KK Manjili MH Baek A Damle SR. DNA methyltransferase inhibition increases efficacy of adoptive cellular immunotherapy of murine breast cancer. Cancer Immunol Immunother (2016) 65(9):1061–73. 10.1007/s00262-016-1868-8 PMC4996686 27416831

[217] Lisiero DN Soto H Everson RG Liau LM Prins RM. The histone deacetylase inhibitor, LBH589, promotes the systemic cytokine and effector responses of adoptively transferred CD8+ T cells. J Immunother Cancer (2014) 2:8. 10.1186/2051-1426-2-8 25054063 PMC4105687

[218] Vo DD Prins RM Begley JL Donahue TR Morris LF Bruhn KW. Enhanced antitumor activity induced by adoptive T-cell transfer and adjunctive use of the histone deacetylase inhibitor LAQ824. Cancer Res (2009) 69(22):8693–9. 10.1158/0008-5472.CAN-09-1456 PMC2779578 19861533

[219] Rao M Chinnasamy N Hong JA Zhang Y Zhang M Xi S. Inhibition of histone lysine methylation enhances cancer-testis antigen expression in lung cancer cells: implications for adoptive immunotherapy of cancer. Cancer Res (2011) 71(12):4192–204. 10.1158/0008-5472.CAN-10-2442 PMC3116976 21546573

[220] Peng D Kryczek I Nagarsheth N Zhao L Wei S Wang W. Epigenetic silencing of TH1-type chemokines shapes tumour immunity and immunotherapy. Nature (2015) 527(7577):249–53. 10.1038/nature15520 PMC4779053 26503055

[221] Kagoya Y Nakatsugawa M Yamashita Y Ochi T Guo T Anczurowski M. BET bromodomain inhibition enhances T cell persistence and function in adoptive immunotherapy models. J Clin Invest (2016) 126(9):3479–94. 10.1172/JCI86437 PMC5004946 27548527

[222] Fraietta JA Nobles CL Sammons MA Lundh S Carty SA Reich TJ. Disruption of TET2 promotes the therapeutic efficacy of CD19-targeted T cells. Nature (2018) 558(7709):307–+. 10.1038/s41586-018-0178-z PMC6320248 29849141

[223] Zebley CC Gottschalk S Youngblood B. Rewriting History: Epigenetic Reprogramming of CD8(+) T Cell Differentiation to Enhance Immunotherapy. Trends Immunol (2020) 41(8):665–75. 10.1016/j.it.2020.06.008 PMC7395868 32624330

[224] Li S Xue L Wang M Qiang P Xu H Zhang X. Decitabine enhances cytotoxic effect of T cells with an anti-CD19 chimeric antigen receptor in treatment of lymphoma. Onco Targets Ther (2019) 12:5627–38. 10.2147/OTT.S198567 PMC6635897 31372000

[225] Kailayangiri S Altvater B Lesch S Balbach S Gottlich C Kuhnemundt J. EZH2 Inhibition in Ewing Sarcoma Upregulates GD2 Expression for Targeting with Gene-Modified T Cells. Mol Ther (2019) 27(5):933–46. 10.1016/j.ymthe.2019.02.014 PMC6520468 30879952

[226] Avanzi MP Yeku O Li X Wijewarnasuriya DP van Leeuwen DG Cheung K. Engineered Tumor-Targeted T Cells Mediate Enhanced Anti-Tumor Efficacy Both Directly and through Activation of the Endogenous Immune System. Cell Rep (2018) 23(7):2130–41. 10.1016/j.celrep.2018.04.051 PMC5986286 29768210

[227] Li X Daniyan AF Lopez AV Purdon TJ Brentjens RJ. Cytokine IL-36gamma improves CAR T-cell functionality and induces endogenous antitumor response. Leukemia (2020) 35(2):506–21. 10.1038/s41375-020-0874-1 PMC7680719 32447345

[228] Walsh SR Simovic B Chen L Bastin D Nguyen A Stephenson K. Endogenous T cells prevent tumor immune escape following adoptive T cell therapy. J Clin Invest (2019) 129(12):5400–10. 10.1172/JCI126199 PMC6877330 31682239

[229] Liu E Marin D Banerjee P Macapinlac HA Thompson P Basar R. Use of CAR-Transduced Natural Killer Cells in CD19-Positive Lymphoid Tumors. N Engl J Med (2020) 382(6):545–53. 10.1056/NEJMoa1910607 PMC7101242 32023374

[230] De Keersmaecker B Claerhout S Carrasco J Bar I Corthals J Wilgenhof S. TriMix and tumor antigen mRNA electroporated dendritic cell vaccination plus ipilimumab: link between T-cell activation and clinical responses in advanced melanoma. J Immunother Cancer (2020) 8(1):e000329. 10.1136/jitc-2019-000329 32114500 PMC7057443

[231] Wilgenhof S Corthals J Heirman C van Baren N Lucas S Kvistborg P. Phase II Study of Autologous Monocyte-Derived mRNA Electroporated Dendritic Cells (TriMixDC-MEL) Plus Ipilimumab in Patients With Pretreated Advanced Melanoma. J Clin Oncol (2016) 34(12):1330–8. 10.1200/JCO.2015.63.4121 26926680

[232] Wall DA Krueger J. Chimeric antigen receptor T cell therapy comes to clinical practice. Curr Oncol (2020) 27(Suppl 2):S115–S23. 10.3747/co.27.5283 PMC7193999 32368181

[233] Rodriguez Perez A Campillo-Davo D Van Tendeloo VFI Benitez-Ribas D. Cellular immunotherapy: a clinical state-of-the-art of a new paradigm for cancer treatment. Clin Transl Oncol (2020) 22(11):1923–37. 10.1007/s12094-020-02344-4 32266674

[234] Sharon E Streicher H Goncalves P Chen HX. Immune checkpoint inhibitors in clinical trials. Chin J Cancer (2014) 33(9):434–44. 10.5732/cjc.014.10122 PMC4190433 25189716

[235] Singh AK Chandra N Bapat SA. Evaluation of Epigenetic Drug Targeting of Heterogenous Tumor Cell Fractions Using Potential Biomarkers of Response in Ovarian Cancer. Clin Cancer Res (2015) 21(22):5151–63. 10.1158/1078-0432.CCR-15-0505 26130461

[236] Noberini R Robusti G Bonaldi T. Mass spectrometry-based characterization of histones in clinical samples: applications, progresses, and challenges. FEBS J (2021). 10.1111/febs.15707 PMC9291046 33415821

[237] Maio M Covre A Fratta E Di Giacomo AM Taverna P Natali PG. Molecular Pathways: At the Crossroads of Cancer Epigenetics and Immunotherapy. Clin Cancer Res (2015) 21(18):4040–7. 10.1158/1078-0432.CCR-14-2914 26374074

[238] Morel D Jeffery D Aspeslagh S Almouzni G Postel-Vinay S. Combining epigenetic drugs with other therapies for solid tumours - past lessons and future promise. Nat Rev Clin Oncol (2020) 17(2):91–107. 10.1038/s41571-019-0267-4 31570827

[239] Oleksiewicz U Machnik M. Causes, effects, and clinical implications of perturbed patterns within the cancer epigenome. Semin Cancer Biol (2020). 10.1016/j.semcancer.2020.12.014 33359485

[240] Hogg SJ Beavis PA Dawson MA Johnstone RW. Targeting the epigenetic regulation of antitumour immunity. Nat Rev Drug Discov (2020) 19(11):776–800. 10.1038/s41573-020-0077-5 32929243

